# Aurora B‐dependent polarization of the cortical actomyosin network during mitotic exit

**DOI:** 10.15252/embr.202152387

**Published:** 2021-08-24

**Authors:** Nitya Ramkumar, Jigna V Patel, Jannis Anstatt, Buzz Baum

**Affiliations:** ^1^ MRC LMCB UCL London UK; ^2^ Present address: Duke University Durham NC USA; ^3^ Present address: MRC‐LMB Cambridge UK

**Keywords:** Aurora B, polar relaxation, actomyosin network, cytokinesis, cell polarization, Cell Adhesion, Polarity & Cytoskeleton, Cell Cycle

## Abstract

The isotropic metaphase actin cortex progressively polarizes as the anaphase spindle elongates during mitotic exit. This involves the loss of actomyosin cortex from opposing cell poles and the accumulation of an actomyosin belt at the cell centre. Although these spatially distinct cortical remodelling events are coordinated in time, here we show that they are independent of each other. Thus, actomyosin is lost from opposing poles in anaphase cells that lack an actomyosin ring owing to centralspindlin depletion. In examining potential regulators of this process, we identify a role for Aurora B kinase in actin clearance at cell poles. Upon combining Aurora B inhibition with centralspindlin depletion, cells exiting mitosis fail to change shape and remain completely spherical. Additionally, we demonstrate a requirement for Aurora B in the clearance of cortical actin close to anaphase chromatin in cells exiting mitosis with a bipolar spindle and in monopolar cells forced to divide while flat. Altogether, these data suggest a novel role for Aurora B activity in facilitating DNA‐mediated polar relaxation at anaphase, polarization of the actomyosin cortex, and cell division.

## Graphical abstract

Aurora B facilitates DNA‐mediated polar relaxation at anaphase and polarization of the actomyosin cortex to ensure a robust coupling of anaphase spindle elongation and cytokinesis.

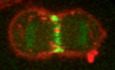

## Introduction

Cell division involves the segregation of genetic material and cellular components into two daughter cells. Cytokinesis is the final step in this process, whereby an actomyosin ring constricts to cleave the cell into two. This process begins with anaphase cell elongation, as spherical cells exiting mitosis adopt an ellipsoid shape. Concomitantly, as the DNA begins to separate and reach the poles, there is a break in the symmetry of the uniform spherical cortical actomyosin network as the cortical actomyosin network softens at the poles and stiffens and constricts at the cell centre. The timely execution of this process requires extensive crosstalk between the spindle and the overlying cortical actomyosin network and is essential for the accurate segregation of sister chromatids at division (Ramkumar & Baum, 2016).

Multiple pathways have been shown to be involved in this crosstalk and in the regulation of cytokinesis in both time and space. Although these events differ between cell types and across evolution, pioneering work from Ray Rappaport provided a crucial framework for understanding this process (Rappaport, 1996). He demonstrated that overlapping antiparallel microtubule from opposing spindle poles position the cleavage furrow—helping to ensure that the actomyosin ring bisects the spindle.

As this contractile actomyosin ring is specified, anaphase cells elongate along their long cell axis. This change in shape has often been attributed to the effects of the gradient in cortical contractility induced via actomyosin ring formation (Pollard, 2010; Turlier *et al*, 2014). However, it has long been appreciated that polar relaxation itself may play a critical part in this process (Wolpert, 1960). In fact, the softening of cell poles is a likely pre‐requisite for the division of animal cells which have undergone prior cortical stiffening during the process of mitotic rounding (Taubenberger *et al*, 2020). In addition, polar re‐spreading has been shown to be sufficient to drive cell division in some cells that lack contractile components of the cleavage furrow (Kanada *et al*, 2005; Dix *et al*, 2018). While the pathways involved in this process of polar relaxation are not yet clear, recent work has suggested a role for DNA‐dependent cues in polar relaxation (Kiyomitsu & Cheeseman, 2012; Rodrigues *et al*, 2015). These data point towards a role for the advancing chromosomes in softening cell poles in early anaphase as an aid to division. Two pathways have been suggested to regulate polar relaxation. A chromatin‐based Ran‐GTP gradient (Kiyomitsu & Cheeseman, 2012) has been proposed to act via importin to limit the accumulation of Anillin at cell poles. In addition, a kinetochore‐associated pool of Sds22/PP1 has been proposed to dephosphorylate cortical ERM proteins (Rodrigues *et al*, 2015). Since ERM proteins link actin cytoskeleton to the membrane, localized ERM dephosphorylation at cell poles was proposed to de‐couple the actomyosin network from the plasma membrane to reduce the mechanical rigidity of the actomyosin network at the poles (Kunda *et al*, 2012). The activation of Arp2/3 at the polar cortex could assist this process by driving polar cell re‐spreading in some systems (Kanada *et al*, 2005; Dix *et al*, 2018).

Due to the dynamic nature of the changes in cell shape and cytoskeletal organization during mitotic exit, it is important that these spatially distinct processes—polar relaxation, contractile ring specification and furrowing, be coordinated. While it is not understood precisely how this is achieved, Aurora B, the core member of the chromosome passenger complex (CPC), likely plays a role in this process. It has been implicated in multiple events at mitotic exit (Carmena *et al*, 2015; Afonso *et al*, 2016; Liu & Pellman, 2020), including in cell and nuclear division (Carmena *et al*, 2015; Petersen & Hagan, 2003; Afonso *et al*, 2014; Roubinet *et al*, 2021), and in spindle assembly checkpoint function (Krenn & Musacchio, 2015). Midzone localized Aurora B has been shown to regulate the oligomerization and accumulation of centralspindlin proteins, which guide the construction of the contractile ring during cytokinesis (Basant *et al*, 2015; Adriaans *et al*, 2019), and to assist in the final steps of abscission (Guse *et al*, 2005; Ahonen *et al*, 2009; Carmena, 2012). In addition, the kinase appears to function at a distance, since a gradient of Aurora B phosphorylation is thought to regulate the separation of DNA during anaphase and to guide the progressive formation of the nuclear envelope soon after (Fuller *et al*, 2008; Afonso *et al*, 2014; Liu & Pellman, 2020). The ability of Aurora B to function in so many distinct processes is likely due to its dynamic localization. Thus, at the metaphase‐anaphase transition, Aurora B undergoes a sudden change in localization from centromeres to the developing spindle midzone and the equatorial cortex (Van Der Horst & Lens, 2014). It is clear from these studies that, despite system specific differences in its function, Aurora B helps to choreograph many of the dramatic changes in cell organization that accompany mitotic exit.

In this paper we identify an additional role for Aurora B in polar relaxation in human cells leaving mitosis. Strikingly, this appears to be independent of Aurora B’s role in midzone stabilization and centralspindlin activity, and appears to be based on a requirement of Aurora B for chromatin‐based cortical remodelling during anaphase. These data lead us to propose that Aurora B coordinates the softening of the cortical cytoskeleton at cell poles close to advancing chromatin and the enhancement of midzone actomyosin ring formation and contraction to ensure a robust coupling of anaphase spindle elongation and cytokinesis.

## Results and Discussion

**Figure 1:**
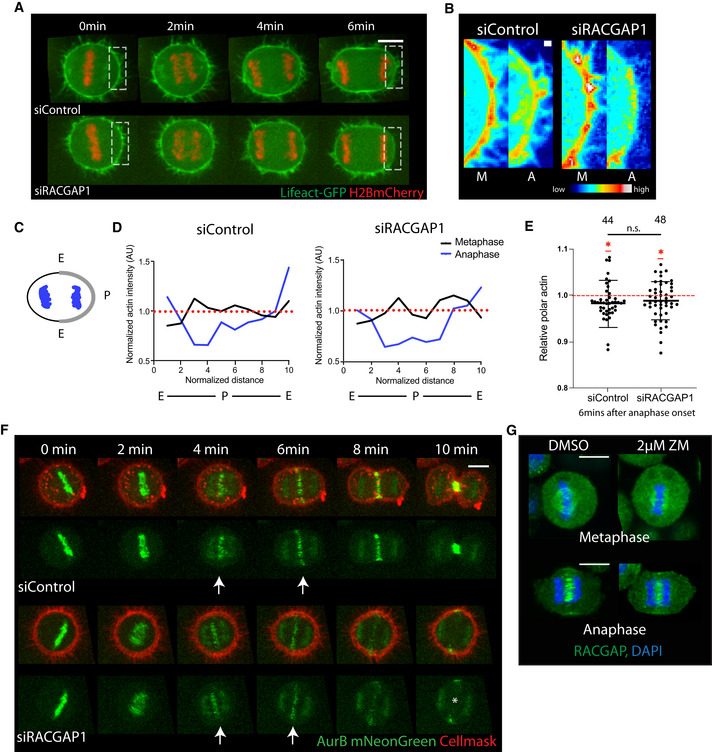
Actin clearance and dynamic re‐localization of Aurora B in early anaphase upon centralspindlin depletion Stills from time‐lapse sequence of representative Hela cells expressing LifeAct GFP and H2B‐mCherry exiting mitosis under different conditions, i.e. Control siRNA and knockdown of the centralspindlin protein RACGAP1. Scale bar = 10 µm.High magnification view of boxed regions in (A) pseudo‐coloured to show actin depletion in anaphase. Scale bar = 1 µm.Schematic representation for quantifying actin intensity along the cortex as in (D). Segmented line was drawn along the cortex from one equatorial region to the other. Actin intensity across the line is plotted in (D).Quantification of actin intensity profile of representative siControl and siRACGAP1‐treated cells shown in (A). While actin levels were uniform across the cortex at metaphase (black line), actin is depleted from the polar regions (Blue line—segments 2–8) of both siControl and siRACGAP1‐treated cells in early anaphase.Quantification of average actin intensity at the poles of cells at 6 min post‐anaphase onset under different conditions as in (A). Despite reduced elongation, actin is cleared from the poles in both Control cells and following RACGAP1 depletion. Data represented as mean ± SD. siControl‐*n* = 44, 0.98 ± 0.051, siRACGAP1‐*n* = 48, 0.99 ± 0.041. Unpaired Welch’s *t*‐test comparing siControl and siRACGAP1 shows no significant difference between them, *P* = 0.5199. One sample *t*‐test comparing siControl and siRACGAP1 to theoretical mean 1 (implying no change in actin compared with metaphase levels) shows significance for both treatments, with *P = 0.0195 for siControl and *P = 0.0392 for siRACGAP1. The depletion seen for both treatments, as means are lower than 1 (red dashed line), suggests significant clearance of actin from cell poles.Stills from time‐lapse sequence of representative Hela cells exiting mitosis, expressing mNeonGreen Aurora B and Cell Mask to label the plasma membrane, showing the dynamic re‐localization of Aurora B from DNA to the overlapping microtubules and cleavage furrow in control siRNA cells (arrows top). Following RACGAP1 silencing, Aurora B still re‐localizes from the DNA to the microtubules and furrow in the midzone in early anaphase (arrows bottom), but the microtubule localization is lost at later stages (asterisk bottom). Scale bar = 10 µm.Maximum projection of 2 z slices of Hela‐Cdk1as cells stained with anti‐RACGAP1 antibody when treated with DMSO or 2 µM ZM447439 (ZM) at metaphase and early anaphase, showing that RACGAP1 relocates to the spindle midzone in early anaphase even upon Aurora B inhibition. Scale bar = 10 µm.

**Figure EV1:**
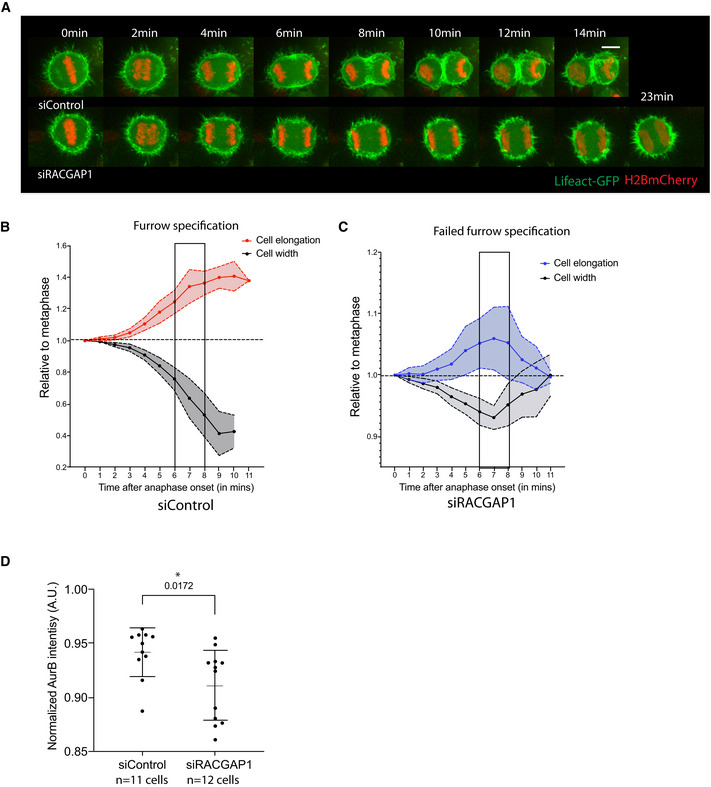
Mitotic exit progression upon centralspindlin depletion Stills from time‐lapse sequence of representative Hela cells expressing LifeAct GFP and H2B‐mCherry exiting mitosis upon Control siRNA treatment or following siRNA‐mediated silencing of the centralspindlin protein RACGAP1 (extension from Fig 1A). Maximum projection of 10 z slices around the middle of the cells shown in Fig 1A. While control‐treated cells form an actomyosin contractile ring that completes ingression by 10 min, upon RACGAP1 depletion cells fail to furrow and remain binucleate. Scale bar = 10 µm.Quantification of cell shape changes—Cell elongation and compression (cell width) in siControl (B) and siRACGAP1‐treated cells (C). siControl‐treated cells begin elongation and midzone flattening at anaphase onset while the cleavage furrow is still being specified. Following furrow specification, around 6–8 min after anaphase onset, the rate of shape changes is enhanced. Average track for 10 representative cells, data are presented as mean ± SD.In siRACGAP1‐treated cells, moderate shape changes are initiated at anaphase onset, seen by increase in cell length and compression of cell. However, unlike in control cells, these cells fail to specify a furrow and therefore these changes are not enhanced and cells begin re‐spreading. Plot shows average track for 10 representative cells. Data are presented as mean ± SD.Quantification of Aurora B levels in the spindle midzone in siControl and siRACGAP1‐treated cells as in Fig 1. Levels were normalized with respect to metaphase levels for each cell. There was a moderate reduction in Aurora B levels at the midzone upon RACGAP1 silencing. Data are represented as mean ± SD, siControl—0.94 ± 0.023, siRACGAP1—0.91 ± 0.033. Unpaired *t*‐test comparing siControl and siRACGAP1, *P = 0.0172.

Centralspindlin proteins are key players in regulating cytokinesis (White & Glotzer, 2012). In human cells, a heterotetrametric complex consisting of two molecules of the GTPase activating protein, RACGAP1, and two molecules of the kinesin, MKLP1, are recruited to the overlapping microtubules of the spindle midzone where they stabilize the midzone and recruit ECT2, a conserved GEF that activates RhoA to drive the local accumulation of contractile actomyosin necessary for cytokinesis (Su *et al*, 2011; Basant & Glotzer, 2018). To assess the relative timing of contractile ring assembly and polar actin clearance during mitotic exit, we performed live cell imaging of cells depleted for RACGAP1 using previously validated siRNAs and a HeLa cell line stably expressing LifeAct GFP and H2B‐mCherry. Cells begin to change shape at anaphase onset. As they begin exiting mitosis, control cells elongate along the spindle axis and constrict in width along a perpendicular axis while the cleavage furrow is still being specified in early anaphase (Fig 1A). These shape changes are further enhanced following the assembly and constriction of the cleavage furrow (Fig EV1A and B). At early stages of anaphase, these changes in cell shape are accompanied by a modest but significant clearance of actin filaments from cell poles (Fig 1A–E), as seen by average actin intensity levels dipping below 1 (normalized to metaphase levels). We observed a similar clearance of LifeAct from the poles of RACGAP1 siRNA‐treated Hela cells exiting mitosis (Fig 1A–E), which exhibit moderate cell elongation and midzone flattening at early anaphase (Fig EV1A and C). This was the case even though RACGAP1 depleted anaphase cells show no sign of assembling an actomyosin ring (Figs 1A, EV1A–C, and 4A and B), eventually leading to division failure (Fig EV1A and C).

These observations show that polar actin clearance is not an indirect consequence of midzone actomyosin ring formation. Instead, the data support the existence of a second pathway that breaks cortical symmetry at the transition from spherical metaphase to elongated anaphase cells (Kiyomitsu & Cheeseman, 2012; Rodrigues *et al*, 2015). This led us to search for additional regulators that might cooperate with centralspindlin proteins in the polarization of the mitotic cortical actomyosin network. For this analysis, we focussed on Aurora B kinase, as it is a conserved regulator of cytokinesis that functions as part of the chromosome passenger complex (Carmena *et al*, 2015; Afonso *et al*, 2016).

To follow Aurora B dynamics, we endogenously tagged Aurora B with mNeonGreen in HeLa cells using CRISPR/Cas9 and imaged these cells as they progressed through mitosis, using red CellMask as a marker of the plasma membrane. As observed previously, Aurora B localizes to centromeres at metaphase in control HeLa cells. Following satisfaction of spindle assembly checkpoint (SAC), Aurora B then re‐localizes to overlapping microtubules within the spindle midzone and to the cleavage furrow (Fig 1F), where it remains to aid abscission (Steigemann *et al*, 2009). In line with previous reports showing that Aurora B re‐localization is unaffected upon knockdown of MKLP1, another centralspindlin protein (Gruneberg *et al*, 2004), this dynamic pattern of Aurora B re‐localization appeared largely unaltered following the silencing of RACGAP1 using RNAi. Although there was a slight reduction in levels of Aurora B::mNeonGreen at the midzone upon RACGAP1silencing (Fig EV1D), the kinase re‐localized from the DNA to midzone microtubules and cleavage furrow in early anaphase with similar timing in both RACGAP1 RNAi and control cells (Fig 1F). Further, Aurora B failed to maintain its later telophase midzone localization in the absence of RACGAP1.

The converse also proved true. Aurora B inhibition did not affect the localization of RACGAP1, as assessed by immunofluorescence, in cells exiting mitosis that had been fixed following treatment with 2 µM of ZM447439 (ZM), a small molecule inhibitor of the Aurora B kinase (Ditchfield *et al*, 2003). Thus, in both control cells and in cells inhibited for Aurora B activity, RACGAP1 was seen localizing to the mitotic spindle in metaphase and to overlapping microtubules within the midzone of the spindle in early anaphase (Fig 1G). Although the midzone localization of RACGAP1 was maintained upon Aurora B inhibition, there was a moderate reduction in its accumulation at the midzone, as seen by increase in cytoplasmic levels. These data suggest that the dynamic re‐localization of Aurora B and RACGAP1 are largely independent of one another in early anaphase HeLa cells.

**Figure 2:**
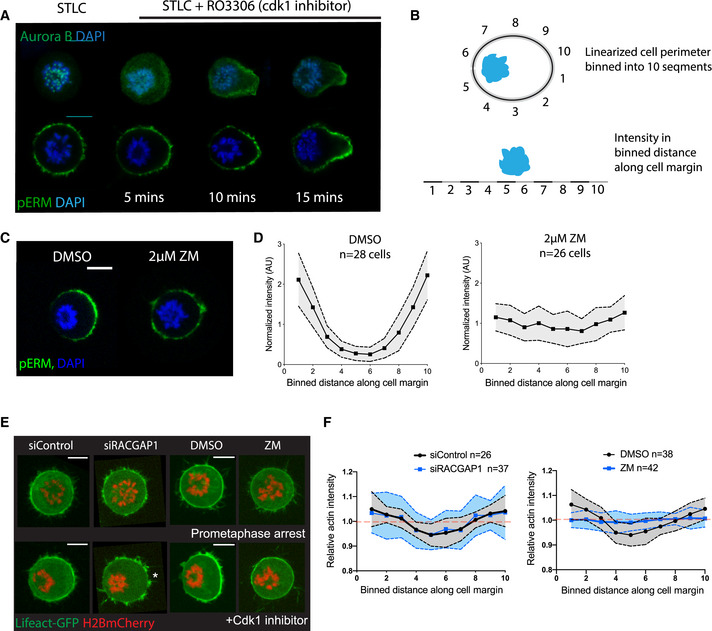
Aurora B is required for polarization of the actomyosin network in the absence of a midzone Maximum projection of 2 z slices of Hela cells immunostained for Aurora B (top) and actin‐membrane cross‐linker pERM (bottom), showing the change in protein localization upon Cdk1 inhibition with RO3306 (exit onset). As DNA moves to one side, Aurora B re‐localizes from DNA to cortical region of the side opposite the DNA. In addition to Aurora B, the membrane‐actin cross‐linker pERM also polarizes in the direction opposite to DNA localization during the course of mitotic exit. Scale bar = 10 µm.Schematic representation of a cell, showing the quantification of actin and pERM along the cortex during mitotic exit. Regions 5 and 6 are cortex close to the DNA, while regions 1 and 10 are the furthest away.Maximum projection of 2 z slices of Hela cells immunostained for pERM following 10 min of forced mitotic exit in presence of DMSO and 2 µM ZM447439. Cells fail to polarize their pERM along the perimeter following Aurora B inhibition, as quantified in (D). Scale bar = 10 µm.Quantification of pERM intensity across the perimeter of the cells as shown in (B) where 5 and 6 represent regions closest to DNA, and 1 and 10 furthest. In DMSO‐treated cells (*n* = 28 cells) pERM levels increase away from the DNA. By contrast, pERM fails to polarize in cells treated with ZM447439 (*n* = 26 cells) during forced mitotic exit. Data are presented as mean ± SD.Stills of representative Hela cells expressing LifeAct GFP and H2B‐mCherry at 10 min following forced mitotic exit with different treatments. Control siRNA, RACGAP1 siRNA and DMSO‐treated cells show clearance of actin from the cortex close to the DNA, while ZM447439‐treated cells fail to clear actin, as quantified in (F). Note, the failure in actin accumulation following RACGAP1 depletion, asterisk. Scale bar = 10 µm.Quantification of actin intensity across the perimeter of the cells, measured based on distance from DNA, with 5 and 6 being closest to DNA and 1 and 10 furthest away, as shown in schematic (B). Graph shows decrease in actin intensity closer to the DNA in siControl (*n* = 26), siRACGAP1 (*n* = 37) and Control DMSO‐treated cells (*n* = 38 cells), seen by average levels lower than 1 (dotted red line), while this local decrease is not seen in ZM447439‐treated cells (*n* = 42 cells). Data are presented as mean ± SD.

To better disentangle the different roles played by Aurora B, it was important to study Aurora B localization and the impact of Aurora B inhibition during the very early part of anaphase in cells that lacked a functional midzone. To do so, we forced cells to exit mitosis with a monopolar spindle, i.e. in the absence of overlapping microtubules by treating cells arrested in prometaphase using STLC, an Eg5 inhibitor (Skoufias *et al*, 2006) with a Cdk1 inhibitor (RO3306) (Canman *et al*, 2003; Hu *et al*, 2008). During this “monopolar cytokinesis”, Aurora B was observed re‐localizing from centromeres to the cell cortex most distant from anaphase chromatin as the DNA moved poleward (Fig 2A), as previously reported (Canman *et al*, 2003). This was coincident with the break in cortical actomyosin symmetry. Thus, phosphorylated ERM proteins (pERM‐Fig 2A), which link actin filaments to the cell membrane (Bretscher *et al*, 2002) and have been associated with mitotic cell shape control (Kunda *et al*, 2008, 2012; Rodrigues *et al*, 2015), were lost from the cortex close to the DNA within 5 min of Cdk1 inhibitor addition. The resulting pERM gradient (Fig 2A) strengthened over time so that within ∼15 min of Cdk1 inhibitor addition most of the cells had a well‐defined cortical gradient of phosphorylated ERM localization (Fig 2A–D).

**Figure EV2:**
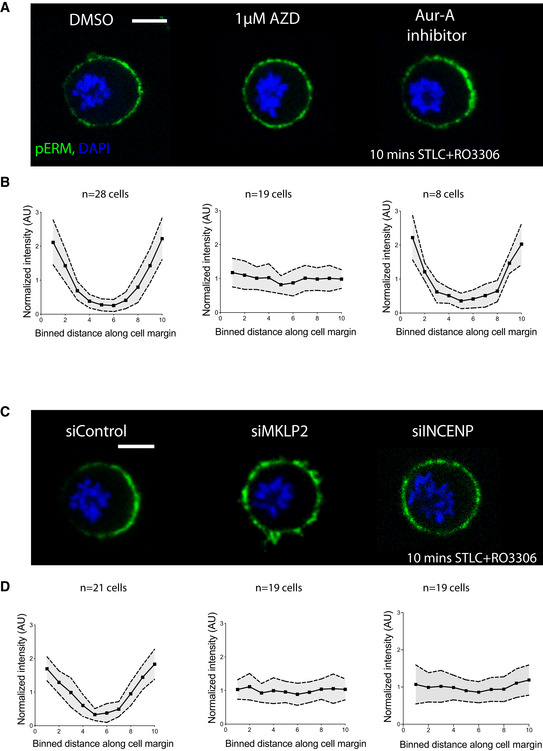
Midzone microtubule‐independent polarization of the cortical actomyosin network by Aurora B during mitotic exit Maximum projection of 2 z slices of Hela cells immunostained for pERM following 10 min of forced mitotic exit in presence of DMSO, 1 µM AZD1152 and 1 µM Aurora A inhibitor I. Cells fail to polarize cortical pERM when treated with another Aurora B inhibitor AZD1152, but are not affected by Aurora‐A inhibition, suggesting that this function is specific to Aurora B during mitotic exit. Scale bar = 10 µm.Quantification of pERM intensity across the perimeter of cells as shown in Fig 2B, where 5 and 6 represent regions closest to DNA, and 1 and 10 furthest. DMSO (*n* = 28 cells) or Aur‐A inhibitor‐treated cells (*n* = 8 cells) display an increase in pERM levels as the distance from the DNA increases, whereas cells treated with AZD1152 (*n* = 19 cells) during exit fail to polarize cortical pERM. Data are represented as mean ± SD.Maximum projection of 2 z slices of Hela cells immunostained for pERM following 10 min of forced mitotic exit after siControl, siINCENP or siKLP2 treatment. While Control siRNA‐treated cells polarize their cortex, depletion of INCENP or MKLP2 leads to a failure in polarization. Note, the clearance of pERM close to DNA in control conditions, which does not occur following INCENP or MKLP2 depletion. Scale bar = 10 µm.Quantification of pERM intensity across the perimeter of the cells shown in (C), where 5 and 6 represent regions closest to DNA, and 1 and 10 furthest. Control siRNA‐treated cells (*n* = 21 cells) show polarization of pERM. By contrast, this polarization is not seen following siRNA‐mediated silencing of INCENP (*n* = 19 cells) or MKLP2 (*n* = 19 cells). Data are represented as mean ± SD.

**Figure EV3:**
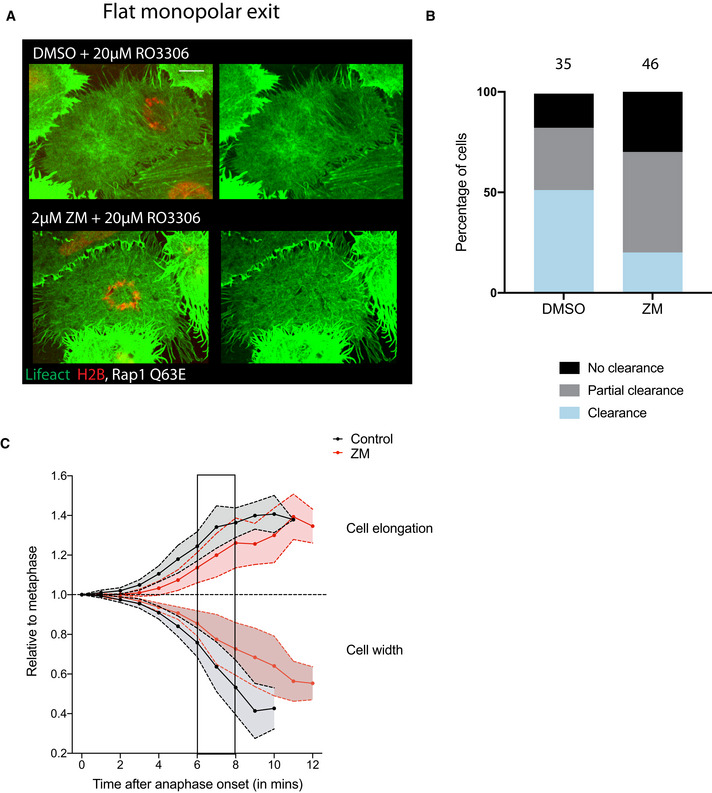
Aurora B activity is required for DNA‐dependent clearance of actin Maximum projection of 2 z slices of the basal most region of Hela cells expressing LifeAct GFP and H2B‐mCherry forced to undergo flat monopolar cytokinesis. This reveals the ability of DNA to clear basal actomyosin at 15 min post‐exit onset, which is lost following Aurora B inhibition with ZM447439 treatment, as quantified in (B). Dotted mask shows position of DNA in the actin channel. Scale bar = 10 µm.Quantification of the fraction of cells in which cortical actin is cleared from underneath the DNA within 15 min of exit onset. 82% of DMSO cells (*n* = 35 cells) clear actin from underneath the DNA (51% completely and 31% partially), whereas only 70% of the cells manage to do so following Aurora B inhibition (20% completely, 50% partially) (*n* = 46 cells). Chi‐square test comparing distribution of cleared vs partial and no clearance shows significant difference between DMSO and ZM treatments, *P* = 0.0026.Quantification of cell shape changes—Cell elongation and midzone flattening and constriction (cell width) in siControl and ZM‐treated cells. siControl‐treated cells begin elongation and midzone flattening in early anaphase. This is further enhanced by furrow constriction. In ZM‐treated cells, the shape changes initiated at early anaphase are slower than those seen in control cells. However, these shape changes are enhanced following furrow specification and constriction, and eventually become comparable to those seen in control cells. Average track for 10 representative cells, control cells track from Fig EV1. Data are represented as mean ± SD.

**Figure EV4:**
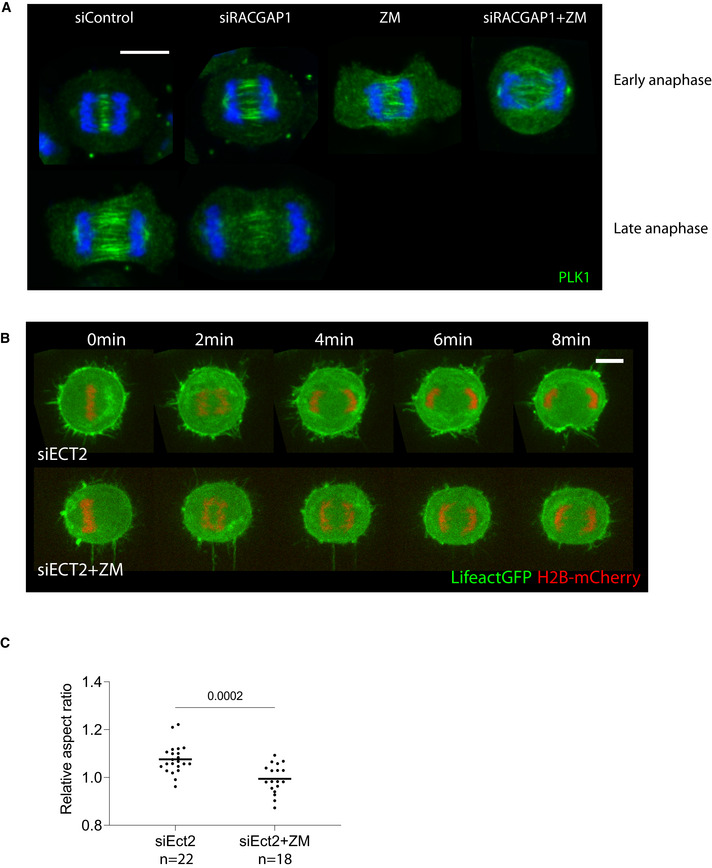
Cooperative effect of Aurora B and centralspindlin on cell shape changes during anaphase Maximum projection of 2 z slices of representative Hela‐Cdk1as cells immunostained for PLK1 which accumulates on spindle midzone with different treatments. Midzone is specified at early anaphase in siRACGAP1‐treated cells, but unlike siControl treated cells, it fails to be stabilized at later stages. In contrast, ZM‐treated cells have a diffuse midzone region during anaphase. In the double treatment condition there is no recognizable midzone region. Scale bar = 10 µm.Stills from time‐lapse of representative Hela cells expressing LifeAct GFP and H2B‐mCherry exiting mitosis. Cells were treated with either siECT2 or siECT2 plus ZM. ECT2 silencing leads to increased cytoplasmic actin and reduced anaphase cell elongation. This effect is further enhanced upon ZM inhibition, which completely abrogates any shape changes. Scale bar = 10 µm.Quantification of cell shape changes during mitotic exit of cells following ECT2 depletion, with or without Aurora B inhibition with ZM, as in (B). While Hela cells with ECT2 depletion undergo some initial shape changes, this is completely abrogated following Aurora B inhibition. Unpaired Welch’s *t*‐test comparing aspect ratio of siECT2‐ and siECT2+ZM‐treated cells shows significant difference between them, *P* = 0.0002. Data are represented as mean ± SD.

**Figure EV5:**
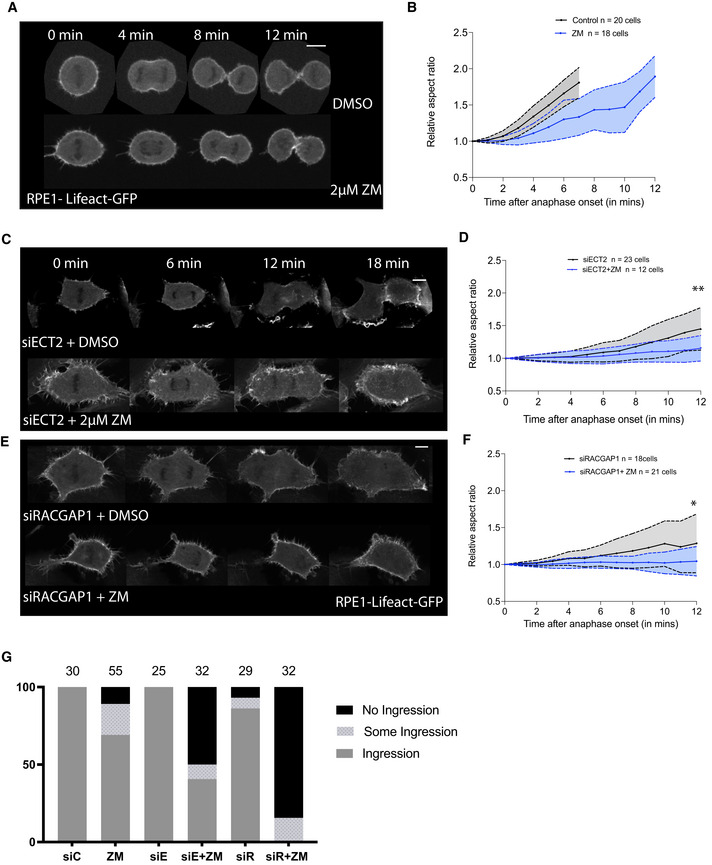
Centralspindlin‐independent function of Aurora B during mitotic exit in RPE1 cells AStills from time‐lapse sequence of RPE1 cells expressing LifeAct GFP, proceeding through anaphase in Control DMSO treated cells and in cells treated with the Aurora B inhibitor, 2 µM ZM. Scale bar = 10 µm.BQuantification of cell shape changes during mitotic exit of cells treated as in (A). Cells are able to furrow upon Aurora B inhibition, but the furrow eventually regresses, leading to the formation of binucleate cells. Note that there is a significant delay in the progression of cell shape changes upon ZM treatment, an indication of a defect in polar relaxation. Data are represented as mean ± SD.CStills from time‐lapse sequence of RPE1 cells expressing LifeAct GFP, treated with siRNAs targetting ECT2 and with DMSO or with ZM, quantified (D). Scale bar = 10 µm.DQuantification of cell shape changes during exit of cells treated as in (C). While cells can divide and change shape following ECT2 depletion (black line), they are unable to even furrow upon ECT2 depletion and Aurora B inhibition (blue line). Note the blue line is centered around 1, suggesting no change in cell shape upon ZM treatment. Unpaired *t*‐test of aspect ratio at 12 min into anaphase comparing siECT2 and siECT2+ZM, **P = 0.0077. Data are represented as mean ± SD.E, FStills from time‐lapse sequence of RPE1 cells expressing LifeAct GFP, treated with siRNA targetting the centralspindlin protein, RACGAP1, treated with DMSO or ZM, quantified in (F). Similar to ECT2, the depletion of RACGAP1 together with Aurora B inhibition leads to a failure in furrow formation and ingression as seen by an average aspect ratio centered around 1 for the double treatment condition (blue line). Data are represented as mean ± SD. Unpaired *t*‐test of aspect ratio at 12 min into anaphase comparing siRACGAP1 and siRACGAP1+ZM, *P = 0.0131. Scale bar = 10 µm.GQuantification of cell shape under conditions described in (A), (C) and (E), showing that depletion of centralspindlin protein RACGAP1 and its downstream effector ECT2 individually have a mild effect on furrow ingression that is compounded when combined with Aurora B inhibition. Chi‐square test comparing distribution of ingressed vs some ingression and no ingression‐ between siControl and ZM treatment, *P* = 0.0007; siECT and siECT2+ZM, *P* < 0.0001 and siRACGAP1 and siRACGAP1+ZM, *P* < 0.0001.

This cortical ERM phosphorylation gradient provided an excellent read‐out of cortical polarization (Fig 2A–C). While pERM accumulated on the cortex furthest from the DNA in control HeLa cells undergoing a monopolar exit, cortical pERM remained isotropic in cells treated with ZM447439, an Aurora B inhibitor (Fig 2C and D). Since Aurora B inhibition also impacts spindle elongation (Hu *et al*, 2008), it was important to measure pERM levels as a function of distance from the DNA in these experiments (Fig 2B). When taking this into account by studying cortical polarization in cells where the DNA succeeded in moving close to one side, pERM still failed to polarize in the absence of Aurora B activity (Fig 2C and D). Furthermore, this function of Aurora B appears specific, since cortical polarization was also compromised in cells exposed to another Aurora B specific inhibitor, AZD1152 (Yang *et al*, 2007) and following RNAi silencing of INCENP, another member of the chromosomal passenger complex (Fig EV2, EV3, EV4, EV5), but was unaffected by the addition of an Aurora A inhibitor (Fig EV2A and B). Interestingly, cortical polarization was affected in a similar way by depletion of MKLP2 (Fig EV2C and D), a kinesin required for Aurora B re‐localization during anaphase (Serena *et al*, 2015). This suggests a role for spatial control of Aurora B activity in polarizing the cortex at mitotic exit.

As a further test of the ability of the DNA‐dependent pathway to induce a local remodelling of the actomyosin cortex in cells lacking Aurora B activity, we imaged cells expressing LifeAct GFP and H2B‐mCherry undergoing monopolar cytokinesis following Aurora B inhibition with ZM447439 treatment. As observed in the context of a bipolar mitosis (Fig 1A–C), actin was cleared from regions of the cortex close to the anaphase chromatin in both control and RACGAP1 siRNA‐treated cells (Fig 2E and F). However, Aurora B inhibition prevented the clearance of actin from areas of the cortex close to the anaphase chromatin in these cells (Fig 2E and F), implying a role for Aurora B activity in DNA‐dependent polarization of cortical actomyosin in anaphase cells.

**Figure 3:**
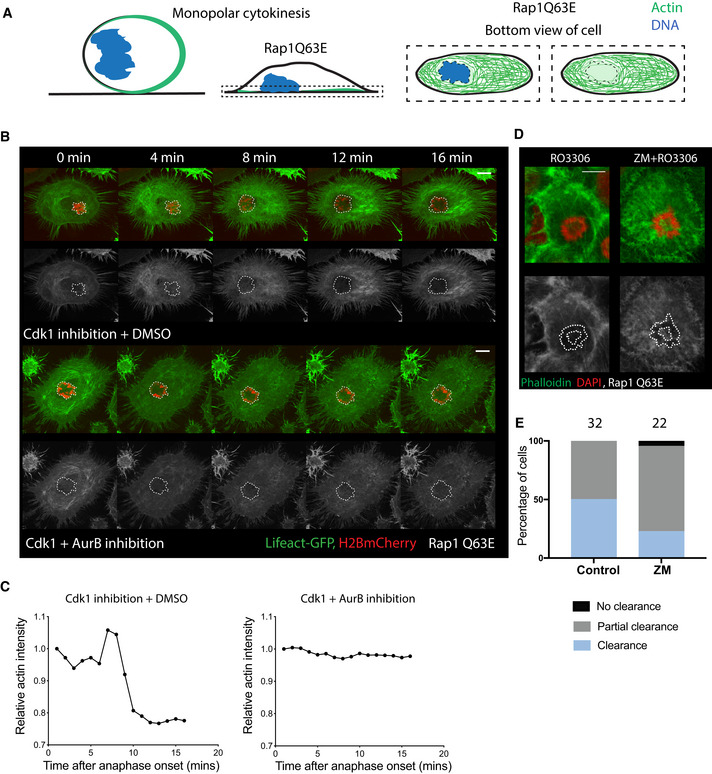
Aurora B inhibition affects DNA‐dependent actin clearance during flat monopolar cytokinesis Schematic of experimental set up. Expression of dominant‐negative Rap1 (RapQ63E) prevents adhesion disassembly during mitotic entry and forces cells to remain flat during mitosis. Using Cdk1 inhibitor to induce monopolar cytokinesis in these STLC‐treated cells, the reduced cell height forces anaphase DNA to come into close proximity with the basal actomyosin network, which we measure to determine the ability of DNA‐dependent cues to relax the local actomyosin network by quantifying actin directly beneath the DNA.Stills from time‐lapse sequence of the basal‐most region of Hela cells expressing LifeAct GFP and H2B‐mCherry forced to undergo flat monopolar mitotic exit. While actin is cleared underneath the DNA in control DMSO‐treated cells, it fails to be cleared around the DNA when cells are treated with Aurora B inhibitor ZM447439 during mitotic exit. Dotted mask shows position of DNA in actin channel. Scale bar – 10 µm.Quantification of clearance of actin underneath the DNA for stills shown in (B). While actin starts to clear from under the DNA in control cells within 10 min of forced mitotic exit, it does not clear following Aurora B inhibition.Maximum projection of 2 z slices of basal most region of Hela cells stained with Phalloidin at 15 min post‐anaphase onset, when cells were treated with DMSO or ZM447439 during flat monopolar cytokinesis, showing clearance under DMSO treatment and a failure to clear actin with ZM447439 treatment. Dotted mask shows position of DNA in actin channel. Scale bar = 10 µm.Quantification of the fraction of cells undergoing local cortical actin clearance during anaphase based on Phalloidin staining as in (D). With DMSO treatment, 50% of the cells clear actin completely, whereas the remaining 50% partially clear actin from beneath the DNA (*n* = 34). Following ZM447439 treatment, only 23% of cells clear local cortical actin completely, while 73% clear it partially, and there is no clearance in 4% of cells (*n* = 22 cells). Chi‐square test comparing distribution of cleared vs partial/no clearance shows significant difference between Control DMSO and ZM treatment, *P* = 0.0413.

Because Aurora B aids the movement of chromatin towards the polar cortex during both bipolar and monopolar exit, we wanted to identify an additional method by which to test the effects of Aurora B inhibition in cells in which the DNA was forced to closely abut the cortex. To do so, we expressed constitutive active Rap1 (Rap1Q63E) to flatten cells undergoing a monopolar cytokinesis. Under these conditions, the DNA was found very close to the basal actomyosin network allowing us to test the ability of DNA‐dependent cue to clear actomyosin during mitotic exit in the presence or absence of Aurora B activity (Fig 3A). In these experiments, the actomyosin network appeared to clear from the basal cortex underlying the DNA within 10 min of Cdk1 inhibition, as measured by the loss of local LifeAct GFP (Fig 3B and C). Importantly, this clearance was prevented by treatment of cells with the Aurora B inhibitor (Fig 3B and C). Furthermore, when we quantified this effect in LifeAct expressing cells 15 min after mitotic exit, actomyosin was cleared from beneath the DNA in 51% of the control cells, while clearance was only observed in 20% of the cells treated with the Aurora B inhibitor (Fig EV3A and B). Similar results were seen when cells were fixed and stained with phalloidin, a marker for filamentous actin. Again, filamentous actin was cleared from the cortex close to the DNA by 15 min of forced mitotic exit in 50% of control DMSO‐treated cells, but was cleared in only 23% of ZM447439‐treated cells (Fig 3D and E). These results suggest that Aurora B activity is required for anaphase chromatin to clear actomyosin network during mitotic exit.

### Cooperative effects of Aurora B and centralspindlin on cortical polarization

The data presented thus far support the possibility of there being two distinct pathways that contribute to polarization of the cortex at anaphase: one that is dependent and one independent of the centralspindlin complex. As a test of this model, we decided to explore how the two systems work when inhibited separately or in concert.

**Figure 4:**
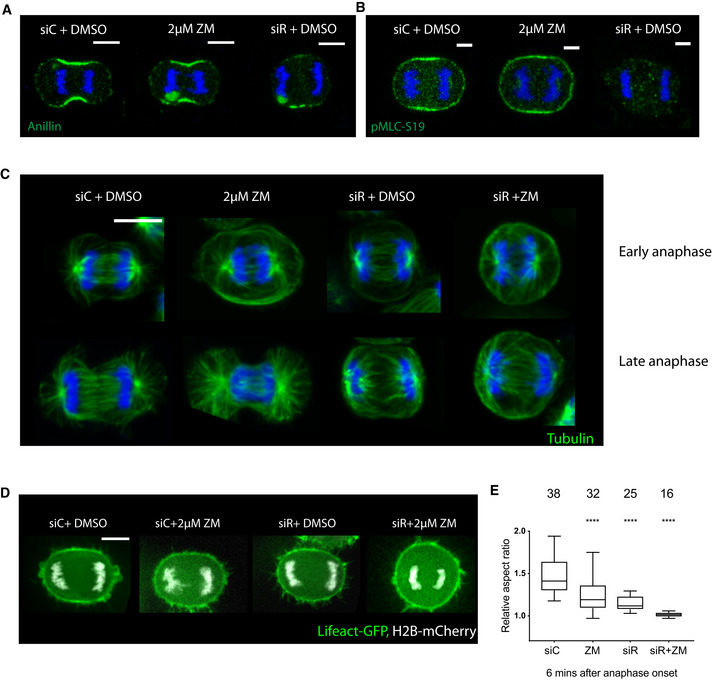
Synergistic effect of Aurora B and centralspindlin during anaphase A, BMaximum projection of 2 z slices of representative Hela cells stained for Anillin (A) and pMLC‐ S19 (B), showing a reduction in these proteins in cells depleted of centralspindlin protein RACGAP1 that is unaltered upon Aurora B inhibition with ZM. Scale bar = 10 µm.CMaximum projection of 2 z slices of representative Hela‐Cdk1as cells treated with siControl, siRACGAP1, 2 µM ZM and siRACGAP1 with 2 µM ZM, immunostained with tubulin to visualize spindle midzone at early anaphase and late anaphase. Midzone is specified in siControl and siRACGAP1‐treated cells, but fails to be maintained in siRACGAP1‐treated cells at later stages. With ZM treatment, a diffuse midzone persists throughout anaphase. No recognizable midzone is seem in the double treatment. Scale bar = 10 µm.DStill images of representative Hela cells expressing LifeAct GFP and H2B‐mCherry captured at early anaphase (6 min after anaphase onset) under different conditions, i.e. Control siRNA, knockdown of centralspindlin protein RACGAP1, in the presence and absence of Aurora B kinase inhibitor (2 µM ZM). Scale bar = 10 µm.EQuantification of aspect ratio of cells treated with different conditions as in (D) at 6 min after anaphase onset, before furrow formation. Individually, Aurora B inhibition and siRACGAP1 have a moderate effect on cell aspect ratio at mitotic exit, while together they have a stronger impact. In the combined treatment, mitotic cells hardly undergo any elongation and remain spherical until they re‐spread sometime later. Ordinary one‐way ANOVA with multiple comparison. Each treatment is compared with siControl, ****P < 0.0001 for all comparisons. Data are represented as box‐whisker plot, with box showing 25^th^–75^th^ percentile values, whiskers‐min to max value and line representing median. Mean ± SD for different treatments‐ siControl (*n* = 38) – 1.455 ± 0.19, ZM (*n* = 32) – 1.219 ± 0.16, siRACGAP1 (*n* = 25) −1.144 ± 0.078 and siRACGAP1+ZM (*n* = 16) – 1.013 ± 0.03. Cells for different treatment pooled from *n* > 3 independent experiments.

On their own, treatments that compromise either Aurora B or centralspindlin had markedly different effects on the equatorial accumulation of downstream cortical cytoskeletal proteins and their regulators (Fig 4A and B). RACGAP1 knockdown led to a complete loss of Anillin and pMLC from the midzone of anaphase cells. Furthermore, RACGAP1 silencing did not prevent midzone specification in early anaphase, as seen by microtubules (Fig 4C) and Plk1 (Fig EV4A) immunostaining, although these patterns of localisation were lost in late anaphase, leading to failure in equatorial accumulation of cytoskeletal proteins. By contrast, Aurora B inhibition had little effect on midzone protein accumulation (Fig 4A and B)—in line with previous reports (Ahonen *et al*, 2009), but led to a dispersed spindle midzone which persisted even at later anaphase stages (Figs 4C and EV4A).

Strikingly, much stronger phenotypes were observed when both pathways were perturbed together. In RACGAP1 RNAi cells treated with ZM, the spindle midzone was not specified at all (Figs 4C and EV4A) and the cells remained completely spherical as they exited mitosis. Similar results were seen using live cell imaging when the two treatments were combined in Hela cells expressing LifeAct‐GFP and H2Bmcherry. While RACGAP1 RNAi and ZM treatment had a modest impact on cell elongation as measured by their aspect ratio prior to furrow formation when studied in isolation (Fig 4D and E), in combination, Aurora B inhibition and RACGAP1 silencing led to a complete loss of anaphase cell shape changes, including cell elongation (Fig 4D and E). A similar effect was seen when Aurora B inhibition was combined with the silencing of Ect2 (Fig EV4B and C)—a downstream target of RACGAP1 and RhoA activator (Yüce *et al*, 2005; Nishimura & Yonemura, 2006).

**Figure 5:**
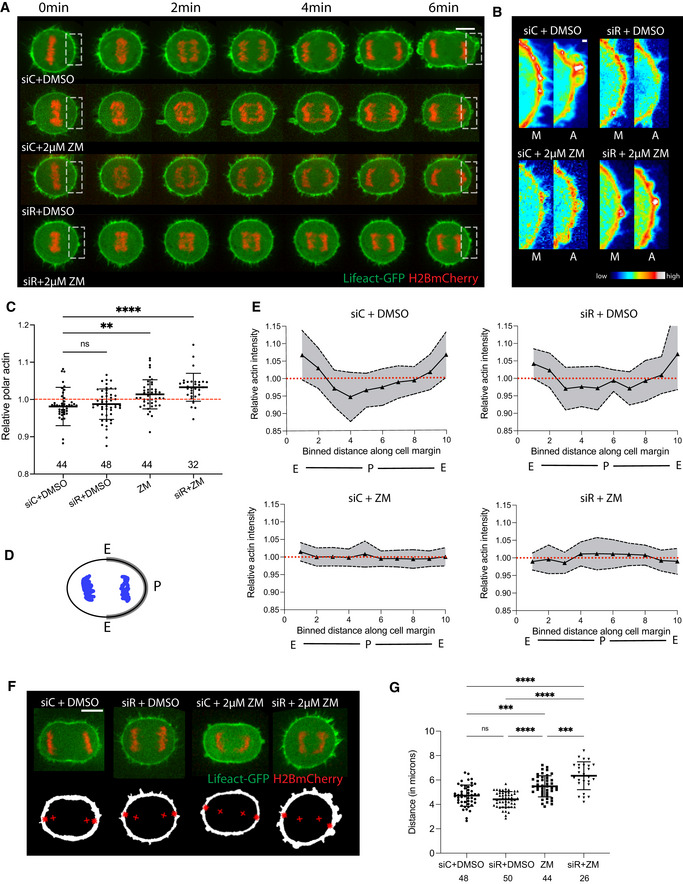
Aurora B inhibition affects DNA‐dependent actin clearance during anaphase Stills from time‐lapse sequence of representative Hela cells expressing LifeAct GFP and H2B‐mCherry, exiting mitosis under different conditions, i.e. Control siRNA, knockdown of centralspindlin protein RACGAP1, in the presence and absence of Aurora B kinase inhibitor (2 µM ZM). Scale bar = 10 µm.High magnification view of boxed regions in (A) pseudo‐coloured showing failure to reduce actin during anaphase in cells treated with Aurora B inhibitor and even greater effect with Aurora B inhibitor upon RACGAP1 depletion. Scale bar = 1 µm.Quantification of actin intensity at the poles of cells at 6 min post‐anaphase onset, before furrow formation under different conditions as in (A). Actin fails to clear from cell poles upon Aurora B kinase inhibition during mitotic exit and this effect is stronger when both centralspindlin and Aurora B kinase activity are affected, while polar actin is cleared upon RACGAP1 depletion. Data are represented as mean ± SD for treatments—siControl‐ 0.98 ± 0.05, siRACGAP1 – 0.99 ± 0.04, ZM—1 ± 0.039, siRACGAP1+ZM—1 ± 0.037. Ordinary one‐way ANOVA with multiple comparison‐ siControl vs siRACGAP1—n.s., *P* = 0.896, siControl vs ZM—**P = 0.0018, siControl vs siRACGAP1+ZM—****P < 0.0001 and ZM vs siRACGAP1+ZM—*P* = 0.1528.Schematic representation for quantifying actin intensity profile along the cortex as in (E).Quantification of average actin intensity profile for siControl, siRACGAP1, ZM and siRACGAP1+ZM‐treated cells shown in (A). While actin levels reduced from the polar regions in both siControl and siRACGAP1‐treated cells seen by levels lower than 1 (dashed red line) in segments 2–8, they failed to be cleared from ZM and siRACGAP1+ZM treatment conditions. Date are represented as mean ± SD.Stills of representative Hela cells expressing LifeAct GFP and H2B‐mCherry captured at early anaphase under different conditions, i.e. Control siRNA, knockdown of RACGAP1, in the presence and absence of Aurora B kinase inhibitor (2 µM ZM). The masks below were generated for each cell and the shortest distance between the centroid of DNA (+) and the masked cortical actomyosin network (*) was quantified in (G) with MATLAB. Scale bar = 10 µm.Quantification of shortest distance between centroid of DNA and cortical actomyosin network. While chromatin is able to reach close to the cortex in both siControl and siRACGAP1‐treated cells, the distance of chromatin from cortex increases upon ZM treatment and this distance is further enhanced in the double treatment condition. Data are represented as mean ± SD. siControl—4.7 ± 0.87, siRACGAP1—4.4 ± 0.64, ZM—5.47 ± 0.87, siRACGAP1+ZM—6.35 ± 1.14 μm. Ordinary one‐way ANOVA with multiple comparison, siControl vs ZM, ***P = 0.002, siControl vs siRACGAP1, *P* = 0.2823, siControl vs siRACGAP1+ZM, ****P < 0.0001, siRACGAP1 vs siRACGAP1+ZM, ****P < 0.0001, ZM vs siRACGAP1, ****P < 0.0001, ZM vs siRACGAP1+ZM, ***P = 0.0004.

A comparable pattern was observed when we examined the impact of the two pathways on polar actin clearance. Again, cells failed to clear actin from the poles following Aurora B inhibition (Fig 5A–E), without visibly perturbing the initial assembly of the actomyosin‐based equatorial furrow (Fig 4A and B). As a result, there was a significant delay in the timing of anaphase cell elongation in cells treated with Aurora B inhibitor relative to control cells (Fig EV3C), suggestive of a defect in polar relaxation. These cells eventually elongated to a similar extent to control cells as they underwent cytokinesis.

The defect in polar anaphase actin clearance was further enhanced by the failure of anaphase chromatin to reach close to the actomyosin cortical network following Aurora B inhibition; and was accentuated by the RACGAP1 knockdown (Fig 5F and G). This failure in polar relaxation upon Aurora B inhibition is likely due to a weaker chromatin‐based signal coupled with a reduction in the elongation of the anaphase spindle. By contrast, RACGAP1 had no significant effect on the DNA‐cortex distance compared with control siRNA‐treated cells and actin was cleared from the poles during both monopolar and bipolar mitosis (Figs 1A–C, 2E and F, and 5A–E). We do not think that the lack of a robust penetrant phenotype reflects an incomplete depletion of RACGAP1, as we observed a complete loss of cortical myosin accumulation and furrow formation in RACGAP1 silenced cells (Fig 4B).

We observed a similar synergistic effect of combining treatments perturbing both RACGAP1 and Aurora B in human RPE1 cells (Fig EV5). Here again, the inhibition of Aurora B led to significant delay in cell elongation at anaphase, but did not affect furrow formation (even though furrows eventually later regressed (Fig EV5A and B)). While RPE1 cells can perform cytokinesis even in the absence of the midzone pathway (Fig EV5C) (Dix *et al*, 2018), when combined with Aurora B inhibition, the silencing of the centralspindlin protein RACGAP1 (Fig EV5E and F) or its downstream target ECT2 (Fig EV5C and D), these cells were unable to undergo cell shape changes at anaphase and were unable to form a cytokinetic furrow (Fig EV5C–G). The data further support the idea that these two pathways function in parallel during cytokinesis.

In summary, in this study we provide evidence supporting a role for Aurora B activity in the disassembly of the polar cortical actomyosin network during early anaphase that is independent of its role in central spindle formation and in the assembly of a contractile actomyosin ring. Further, our data suggest that Aurora B does so by facilitating the DNA‐dependent signal that previous work has implicated in polar relaxation.

While important in human cells in culture, which have a large spindle that comes into close contact with the cortex, the importance and mechanism of polar relaxation for successful completion of cytokinesis is likely to be cell type or organism dependent. For instance, in the *Drosophila* notum, cells which fail to undergo polar relaxation complete cytokinesis, although these cells exhibit defects during the process (Rodrigues *et al*, 2015). Similarly, in Hela cells, a failure to undergo polar relaxation leads to instabilities in the contractile ring, cell shape oscillations, but only sometimes to a failure in cytokinesis (Sedzinski *et al*, 2011). Conversely, while most RPE1 cells are able to divide using adhesion‐dependent polarization without the centralspindlin complex or ECT2 function, they often fail to furrow when treated with Aurora B inhibitor in concert. Furthermore, when both pathways are compromised together, the vast majority of cells fail to divide (Fig EV5G). Although Aurora B may play a similar function in other systems, we think this will depend on the geometrical constraints involved. Thus, we think Aurora B is unlikely to polarize the cortex in *C. elegans* zygotes, since the poles of the anaphase spindle never approach close to the cortex in these cells.

Although we did not observe a defect in DNA‐dependent clearance upon Aurora A inhibition in human cells (Fig EV2A and B), Aurora A has been identified as an aster‐based signal for facilitating polar relaxation in the *C. elegans* zygote (Mangal *et al*, 2018). Since the two homologous Aurora kinases have a largely similar kinase domain, their function may largely reflect their different dynamic sub‐cellular localization (Fu *et al*, 2009; Li *et al*, 2015).

How might this function of Aurora B in chromatin‐induced polar relaxation work mechanistically? While much more work needs to be done, previous work implicated chromosome localized PP1 and its subunit Sds22 in relaxation of the polar cortical actomyosin network through the dephosphorylation of ERM proteins (Kunda *et al*, 2012; Rodrigues *et al*, 2015). This is relevant as PP1 activity and its localization can be regulated by the activity of its binding partners, and Aurora B has been shown to oppose the activity of PP1 during mitotic exit by phosphorylating the conserved binding motif R‐V‐[S/T]‐F in proteins that bind to and regulate PP1 activity (Nasa *et al*, 2018). Further, this Aurora B‐dependent phosphorylation of regulatory proteins can lead to altered PP1 binding. These data lead us to hypothesize that the change in Aurora B localisation and activity at the metaphase‐anaphase transition facilitates polar relaxation and cytokinesis by helping to alter the localization and/or activity of the kinetochore pool of PP1. This is in line with other data that suggest a requirement for Aurora B activity at the metaphase‐to‐anaphase transition, as blocking Aurora B activity around this transition using INCENP antibody has a severe effect on actomyosin ring contraction, while inhibition at a later stage leads to abnormal midbody formation and defects in abscission (Ahonen *et al*, 2009). Further, our results with flat monopolar cytokinesis indicate that proximity of the chromatin to the actomyosin network is critical for its relaxation, as the chromatin‐based signal can only relax the actomyosin network directly underneath it (Fig 3B and C). Thus, Aurora B could indirectly regulate polar relaxation by regulating spindle elongation, therefore controlling the proximity of the DNA to the cortical actomyosin network. Additionally, chromatin bound Aurora B at mitotic exit could influence polar relaxation through its effects on timely assembly of a nuclear envelope (Liu & Pellman, 2020), which would then insulate the poles from the chromatin signal regulating polar relaxation.

In summary, to generate two cells with an equal complement of cellular material, multiple events across the cell must be tightly coordinated in both space and time as cells undergo cytokinesis. While more remains to be learned about how this is achieved across systems, our data suggest that dynamic changes in Aurora B (and the CPC (Landino & Ohi, 2016; Landino *et al*, 2017)) localization at anaphase help to stabilize midzone microtubules, promote ingression of the furrow and trigger timely polar relaxation. In this way, Aurora B appears to play a key role in the orchestration of mammalian cytokinesis.

## Materials and Methods

### Cell culture, drug treatments and siRNA treatment

HeLa Kyoto cells, Hela‐13 cells (LifeAct GFP, H2B‐mCherry) (Matthews *et al*, 2012) and Hela‐Cdk1as (Ruppert *et al*, 2018) were cultured in DMEM (Gibco) supplemented with 10% foetal bovine serum and penicillin/streptomycin (Gibco) at 37°C and 5% CO_2_. hTERT‐RPE1 (female) cells (Clontech) were cultured in DMEM F‐12 GlutaMax (Gibco 31331‐028), with 10% foetal bovine serum, 3.4% sodium bicarbonate (Gibco 25080‐060), 1% Pen‐Strep (Gibco 15070‐063).

For monopolar cytokinesis, cells were synchronized in prometaphase using 5 µM S‐trityl‐L‐cysteine (STLC; Sigma) for 18 h. Monopolar cytokinesis was initiated by the addition of 20 μM RO‐3306 (Enzo Life Sciences), an inhibitor of Cdk1/CyclinB to STLC‐treated cells. 2 µM ZM447439 (Selleck) and 1 µM AZD1152 (Selleck) were used for Aurora B inhibition and 1 µM of Aurora A inhibitor I (Selleck‐S1451) was used for Aurora A inhibition. For inhibition during monopolar cytokinesis, 2 µM ZM447439 or corresponding drugs were added together with Cdk1 inhibitor.

For siRNA knockdown, HeLa Kyoto, Hela‐13, Hela‐Cdk1as and RPE1 cells were transfected with previously published siRNAs using Lipofectamine RNAiMAX (Invitrogen): RACGAP1 (HSS120934—Invitrogen), MKLP2 (Hs_KIF20A_5—Qiagen), INCENP (Dharmacon M‐006823‐00) and Ect2 (Hs_ECT2_6 FlexiTube siRNA). Following at least 24 h of transfection, cells were used for imaging or fixed for immunostaining. For Hela‐Cdk1as‐ 2 μM 1NM‐PP1 was added to the medium 2 h after transfection for synchronization. Following an incubation period of 18 h, cells were washed with media. They were treated with either DMSO or ZM 2 h after washing and processed for fixed immunostaining.

### Aurora B knock‐in cell line

The protocol was adopted from (Lackner *et al*, 2015). Generic donor plasmids (Tia1l) were kind gift from Tilmann Burckstummer. We replaced Turbo‐GFP with mNeonGreen(Tia1l_mNeon). gRNA (5’caccGAGAAGGAGAACTCCTACCCC3’) was designed against N‐terminus of human Aurora Kinase B (ENSG00000178999) and cloned in pX330‐U6‐Chimeric_BB‐CBh‐hSpCas9 (addgene 42230) without any frame shift.

For transfection, lowest passage HeLa cells were seeded in 12‐well dish in antibiotic‐free media (DMEM+10%FBS) overnight to get 70–80% confluency. pX330‐gRNA (300 ng) and Tia1l_mNeon (200 ng) were diluted in 75 μl Optimen, incubated for 10 min in pre‐made transfection mix (75 μl optimen + 3.1 μl Lipofectamine LTX) and applied to cells. Media was replaced next day to complete media (with antibiotics) and cells were grown for a week. GFP positive cells were selected by flow cytometry followed by single cell cloning. Clones were selected by western blotting and localization was confirmed by live cell imaging.

### Live cell imaging

Hela‐13 stable cell lines expressing LifeAct GFP and histone2B‐mRFP or RPE1 cells expressing LifeAct, were plated on 4‐well Lab‐Tek chamber slides coated with 10 μg/ml fibronectin (Sigma) at least 12 h before imaging. Cells entering metaphase were selected and their positions recorded. Following DMSO or 2 µM ZM447439 addition, cells were re‐focussed and images were acquired every 1 min as cells exited mitosis. For flat monopolar cytokinesis, Hela‐13 cells were plated on Lab‐Tek chamber slides. The following day, they were transfected with *pRK5‐Rap1[Q63E]* using Lipofectamine 2000 according to the manufacturer’s instructions. ∼24 h after transfection, cells were then treated with 5 µM STLC for 6 h. Cells transfected with Rap1 Q63E were identified by their failure to round up in prometaphase and were forced to exit mitosis upon Cdk1 inhibition in presence or absence of Aurora B inhibitor. Cells were either fixed after 15 min or were imaged during the exit process.

### Immunostaining

Cells were incubated with freshly prepared 4%PFA for 20 min at room temperature, or ice cold 10% TCA for 20 min. Following washes with PBS‐0.1% Triton, they were incubated with 5%BSAin PBS as blocking buffer and primary antibody was diluted in 1% BSA/PBS for overnight at 4C. Following washes, the cells were incubated with secondary antibody and DAPI (and Phalloidin) for 1 h at room temperature. The slides were mounted with Prolong Gold. Antibodies used—pERM (Cell Signaling 3726), pMLC (Cell Signaling 3671), RACGAP1 (Everest Biotech), Aurora B (Abcam 2254), anillin (gift from C. Field), PLK1 (Cell Signalling 4513) and tubulin (Sigma T9026).

### Quantification

#### Cell shape parameters

Cells were outlined manually in FIJI from metaphase to 6 min post‐anaphase onset (or till furrow constriction). Timing of anaphase onset was one frame before any visible DNA separation. The aspect ratio was obtained in FIJI and the values were normalized to metaphase for each cell.

#### Monopolar cytokinesis

For actin and pERM along the cell–cell outlines were drawn manually in FIJI on the 2 z slice projection of the midplane region of the cell. The perimeter was straightened and segmented into 10 equal domains, which were numbered based on proximity to DNA, 1 and 10 being furthest away, while 5 and 6 being closest to DNA. The actin/pERM intensity profile along these binned domains was normalized to the cell average and then plotted with respect to binned position along cell using PRISM.

#### Bipolar actin clearance

Polar regions of interest were selected manually on anaphase cells. The actin intensity was measured from 2 z slice projections of midplane region, both at anaphase and a similar region of the cell during metaphase. The relative actin intensity was defined as ratio of actin anaphase:metaphase for each cell, if value of <1 indicates that actin has been cleared while > 1 indicates no clearance.

#### Flat monopolar clearance

DNA signal was used to create a mask, which was replicated in the LifeAct channel. The actin intensity was measured in this masked region in the 2 z slice projection from the bottom region of the cell. Cell outlines were drawn manually and used to determine average actin intensity in the cell. Relative actin intensity was defined by normalizing average actin intensity in masked region (underneath DNA) to average actin intensity in the cell. Value < 1 indicates clearance. For population of cells, we noticed that this ratio was highly variable, owing to the ranging extent of DNA coming in proximity with the basal cortex. Therefore, we obtained the median value of this ratio for the population of cells and classified them as—values between 1 and median as partial clearance and values less than median as complete clearance.

#### DNA‐cortex distance measurements

2 z slice projection of the midplane region of cell was used to create masks for actin (LifeAct) signal with segmentation algorithm in Fiji and the corresponding masked regions isolated from actin channel. Using custom MATLAB code, we calculated the shortest distance from the centroid of each separated DNA to the masked actin channel at early anaphase.

#### Line scan plot

A segmented line of width 10 pixels was drawn on the actin cortex from one equatorial region to the other, encompassing the polar region. 2 z slice projection of the midplane region of cell was used. Actin intensity was measured across the entire line using plot prolife function in Fiji. Values were normalized to metaphase levels for each cell, binned into 10 segments and plotted in Fig 1. For average plots, Fig 5, segmented line was straightened, divided into 10 bins and average intensity in each bin measured. Values were normalized to metaphase levels for each cell and distribution of cell profiles plotted as mean ± SD.

## Author contributions

Conceptualization of project: NR and BB. Design and experiments, and data analysis: NR. Immunostainings in Figs 4 and EV4: JA. CRISPR Aurora B knock‐in cell line: JVP. Manuscript writing: NR and BB.

## Conflict of interest

The authors declare that they have no conflict of interest.

## Supporting information



Expanded View Figures PDF

## Data Availability

This study includes no data deposited in external repositories.
