# Supplementary material for: Aurora B‐dependent polarization of the cortical actomyosin network during mitotic exit
Source: EMBO Rep. 2021 Aug 24;22(10):e52387. doi: 10.15252/embr.202152387 (PMC8490981; doi:10.15252/embr.202152387)
Supplement: Supplementary file 1 — Expanded View Figures PDF [file EMBR-22-e52387-s001.pdf]

Expanded View Figures

Figure EV1. Mitotic exit progression upon centralspindlin depletion.

A Stills from time-lapse sequence of representative Hela cells expressing LifeAct GFP and H2B-mCherry exiting mitosis upon Control siRNA treatment or following siRNA-mediated silencing of the centralspindlin protein RACGAP1 (extension from Fig 1A). Maximum projection of 10 z slices around the middle of the cells shown in Fig 1A. While control-treated cells form an actomyosin contractile ring that completes ingression by 10 min, upon RACGAP1 depletion cells fail to furrow and remain binucleate. Scale bar = 10 μm.

B Quantification of cell shape changes—Cell elongation and compression (cell width) in siControl (B) and siRACGAP1-treated cells (C). siControl-treated cells begin elongation and midzone flattening at anaphase onset while the cleavage furrow is still being specified. Following furrow specification, around 6–8 min after anaphase onset, the rate of shape changes is enhanced. Average track for 10 representative cells, data are presented as mean $\pm$ SD.

C In siRACGAP1-treated cells, moderate shape changes are initiated at anaphase onset, seen by increase in cell length and compression of cell. However, unlike in control cells, these cells fail to specify a furrow and therefore these changes are not enhanced and cells begin re-spreading. Plot shows average track for 10 representative cells. Data are presented as mean $\pm$ SD.

D Quantification of Aurora B levels in the spindle midzone in siControl and siRACGAP1-treated cells as in Fig 1. Levels were normalized with respect to metaphase levels for each cell. There was a moderate reduction in Aurora B levels at the midzone upon RACGAP1 silencing. Data are represented as mean $\pm$ SD, siControl—0.94 $\pm$ 0.023, siRACGAP1—0.91 $\pm$ 0.033. Unpaired *t*-test comparing siControl and siRACGAP1, $^{*}P = 0.0172$.

 © 2021 The Authors

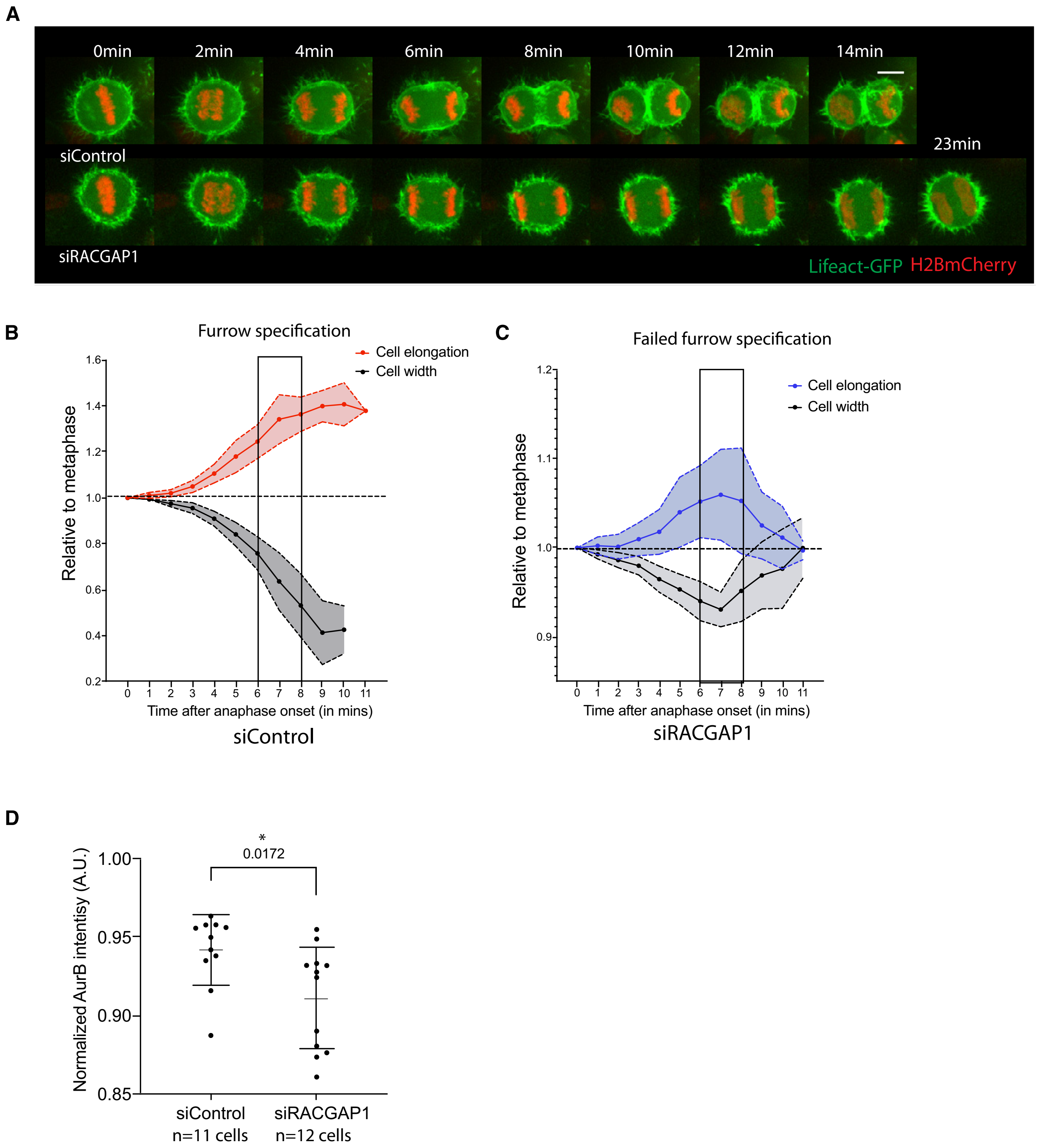
A
0min
2min
4min
6min
8min
10min
12min
14min
23min
siControl
siRACGAP1
Lifeact-GFP H2BmCherry
B
Furrow specification
Cell elongation
Cell width
Relative to metaphase
Time after anaphase onset (in mins)
siControl
C
Failed furrow specification
Cell elongation
Cell width
Relative to metaphase
Time after anaphase onset (in mins)
siRACGAP1
D
*
0.0172
Normalized AurB intentisy (A.U.)
siControl
n=11 cells
siRACGAP1
n=12 cells

Figure EV1.

Figure EV2. Midzone microtubule-independent polarization of the cortical actomyosin network by Aurora B during mitotic exit.

A Maximum projection of 2 z slices of Hela cells immunostained for pERM following 10 min of forced mitotic exit in presence of DMSO, 1 μM AZD1152 and 1 μM Aurora A inhibitor I. Cells fail to polarize cortical pERM when treated with another Aurora B inhibitor AZD1152, but are not affected by Aurora-A inhibition, suggesting that this function is specific to Aurora B during mitotic exit. Scale bar = 10 μm.

B Quantification of pERM intensity across the perimeter of cells as shown in Fig 2B, where 5 and 6 represent regions closest to DNA, and 1 and 10 furthest. DMSO (n = 28 cells) or Aur-A inhibitor-treated cells (n = 8 cells) display an increase in pERM levels as the distance from the DNA increases, whereas cells treated with AZD1152 (n = 19 cells) during exit fail to polarize cortical pERM. Data are represented as mean ± SD.

C Maximum projection of 2 z slices of Hela cells immunostained for pERM following 10 min of forced mitotic exit after siControl, siINCENP or siKLP2 treatment. While Control siRNA-treated cells polarize their cortex, depletion of INCENP or MKLP2 leads to a failure in polarization. Note, the clearance of pERM close to DNA in control conditions, which does not occur following INCENP or MKLP2 depletion. Scale bar = 10 μm.

D Quantification of pERM intensity across the perimeter of the cells shown in (C), where 5 and 6 represent regions closest to DNA, and 1 and 10 furthest. Control siRNA-treated cells (n = 21 cells) show polarization of pERM. By contrast, this polarization is not seen following siRNA-mediated silencing of INCENP (n = 19 cells) or MKLP2 (n = 19 cells). Data are represented as mean ± SD.

 © 2021 The Authors

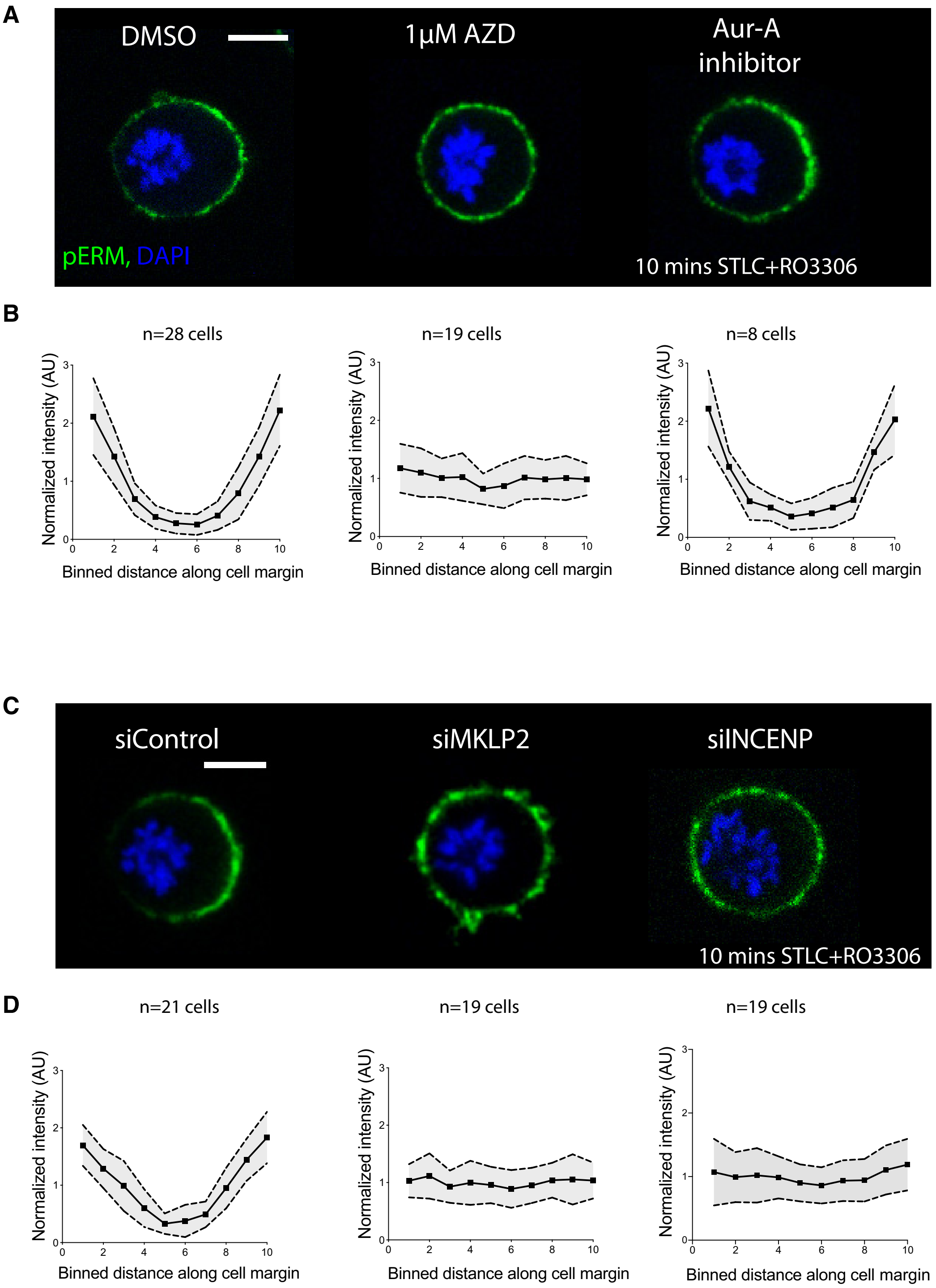

Figure EV2.

© 2021 The Authors

Figure EV3. Aurora B activity is required for DNA-dependent clearance of actin.

A Maximum projection of 2 z slices of the basal most region of Hela cells expressing LifeAct GFP and H2B-mCherry forced to undergo flat monopolar cytokinesis. This reveals the ability of DNA to clear basal actomyosin at 15 min post-exit onset, which is lost following Aurora B inhibition with ZM447439 treatment, as quantified in (B). Dotted mask shows position of DNA in the actin channel. Scale bar = 10 µm.

B Quantification of the fraction of cells in which cortical actin is cleared from underneath the DNA within 15 min of exit onset. 82% of DMSO cells (n = 35 cells) clear actin from underneath the DNA (51% completely and 31% partially), whereas only 70% of the cells manage to do so following Aurora B inhibition (20% completely, 50% partially) (n = 46 cells). Chi-square test comparing distribution of cleared vs partial and no clearance shows significant difference between DMSO and ZM treatments, P = 0.0026.

C Quantification of cell shape changes—Cell elongation and midzone flattening and constriction (cell width) in siControl and ZM-treated cells. siControl-treated cells begin elongation and midzone flattening in early anaphase. This is further enhanced by furrow constriction. In ZM-treated cells, the shape changes initiated at early anaphase are slower than those seen in control cells. However, these shape changes are enhanced following furrow specification and constriction, and eventually become comparable to those seen in control cells. Average track for 10 representative cells, control cells track from Fig EV1. Data are represented as mean ± SD.

 © 2021 The Authors

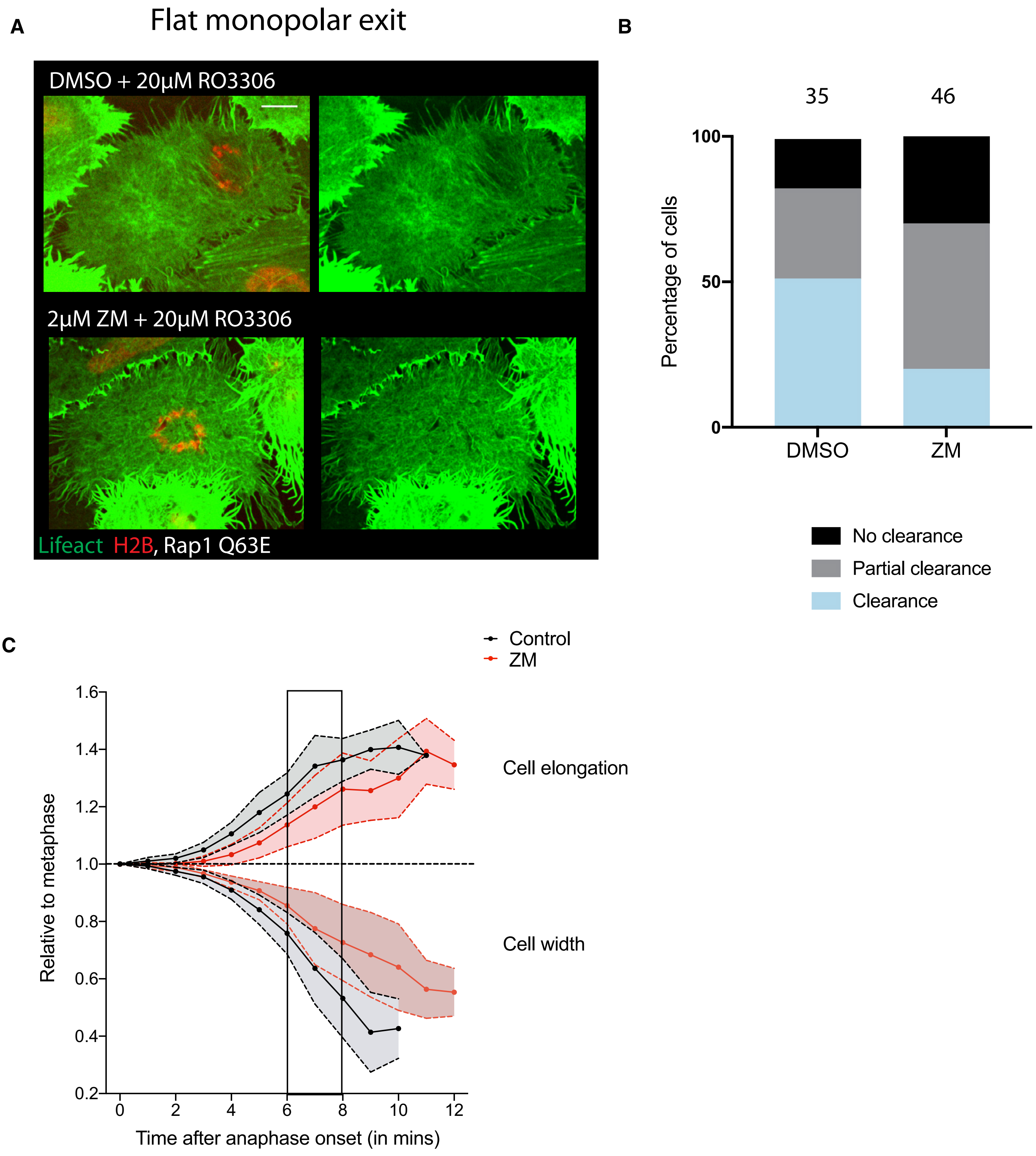
A
Flat monopolar exit
DMSO + 20µM RO3306
2µM ZM + 20µM RO3306
Lifeact H2B, Rap1 Q63E
B
35
46
Percentage of cells
100
50
0
DMSO
ZM
No clearance
Partial clearance
Clearance
C
Control
ZM
Relative to metaphase
1.6
1.4
1.2
1.0
0.8
0.6
0.4
0.2
0
2
4
6
8
10
12
Time after anaphase onset (in mins)
Cell elongation
Cell width

Figure EV3.

Figure EV4. Cooperative effect of Aurora B and centralspindlin on cell shape changes during anaphase.

A Maximum projection of 2 z slices of representative Hela-Cdk1as cells immunostained for PLK1 which accumulates on spindle midzone with different treatments. Midzone is specified at early anaphase in siRACGAP1-treated cells, but unlike siControl treated cells, it fails to be stabilized at later stages. In contrast, ZM-treated cells have a diffuse midzone region during anaphase. In the double treatment condition there is no recognizable midzone region. Scale bar = 10 μm.

B Stills from time-lapse of representative Hela cells expressing LifeAct GFP and H2B-mCherry exiting mitosis. Cells were treated with either siECT2 or siECT2 plus ZM. ECT2 silencing leads to increased cytoplasmic actin and reduced anaphase cell elongation. This effect is further enhanced upon ZM inhibition, which completely abrogates any shape changes. Scale bar = 10 μm.

C Quantification of cell shape changes during mitotic exit of cells following ECT2 depletion, with or without Aurora B inhibition with ZM, as in (B). While Hela cells with ECT2 depletion undergo some initial shape changes, this is completely abrogated following Aurora B inhibition. Unpaired Welch's *t*-test comparing aspect ratio of siECT2- and siECT2+ZM-treated cells shows significant difference between them, $P = 0.0002$. Data are represented as mean ± SD.

© 2021 The Authors

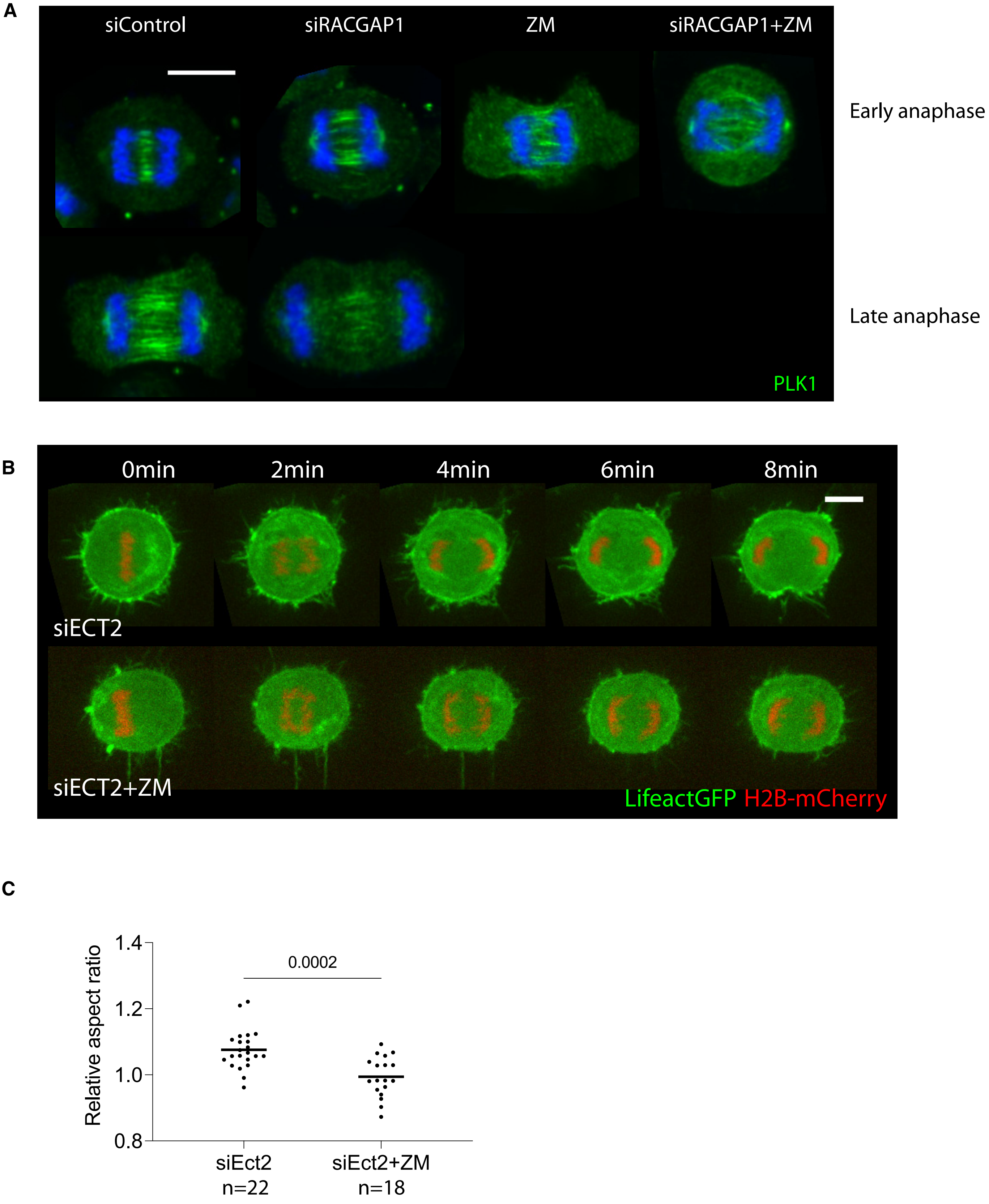
A
siControl
siRACGAP1
ZM
siRACGAP1+ZM
Early anaphase
Late anaphase
PLK1
B
0min
2min
4min
6min
8min
siECT2
siECT2+ZM
LifeactGFP H2B-mCherry
C
Relative aspect ratio
1.4
1.2
1.0
0.8
0.0002
siEct2
n=22
siEct2+ZM
n=18

Figure EV4.

Figure EV5. Centralspindlin-independent function of Aurora B during mitotic exit in RPE1 cells.

A Stills from time-lapse sequence of RPE1 cells expressing LifeAct GFP, proceeding through anaphase in Control DMSO treated cells and in cells treated with the Aurora B inhibitor, 2 μM ZM. Scale bar = 10 μm.

B Quantification of cell shape changes during mitotic exit of cells treated as in (A). Cells are able to furrow upon Aurora B inhibition, but the furrow eventually regresses, leading to the formation of binucleate cells. Note that there is a significant delay in the progression of cell shape changes upon ZM treatment, an indication of a defect in polar relaxation. Data are represented as mean ± SD.

C Stills from time-lapse sequence of RPE1 cells expressing LifeAct GFP, treated with siRNAs targetting ECT2 and with DMSO or with ZM, quantified (D). Scale bar = 10 μm.

D Quantification of cell shape changes during exit of cells treated as in (C). While cells can divide and change shape following ECT2 depletion (black line), they are unable to even furrow upon ECT2 depletion and Aurora B inhibition (blue line). Note the blue line is centered around 1, suggesting no change in cell shape upon ZM treatment. Unpaired *t*-test of aspect ratio at 12 min into anaphase comparing siECT2 and siECT2+ZM, $^{**}P = 0.0077$. Data are represented as mean ± SD.

E, F Stills from time-lapse sequence of RPE1 cells expressing LifeAct GFP, treated with siRNA targetting the centralspindlin protein, RACGAP1, treated with DMSO or ZM, quantified in (F). Similar to ECT2, the depletion of RACGAP1 together with Aurora B inhibition leads to a failure in furrow formation and ingression as seen by an average aspect ratio centered around 1 for the double treatment condition (blue line). Data are represented as mean ± SD. Unpaired *t*-test of aspect ratio at 12 min into anaphase comparing siRACGAP1 and siRACGAP1+ZM, $^{*}P = 0.0131$. Scale bar = 10 μm.

G Quantification of cell shape under conditions described in (A), (C) and (E), showing that depletion of centralspindlin protein RACGAP1 and its downstream effector ECT2 individually have a mild effect on furrow ingression that is compounded when combined with Aurora B inhibition. Chi-square test comparing distribution of ingressed vs some ingression and no ingression- between siControl and ZM treatment, $P = 0.0007$; siECT and siECT2+ZM, $P < 0.0001$ and siRACGAP1 and siRACGAP1+ZM, $P < 0.0001$.

© 2021 The Authors

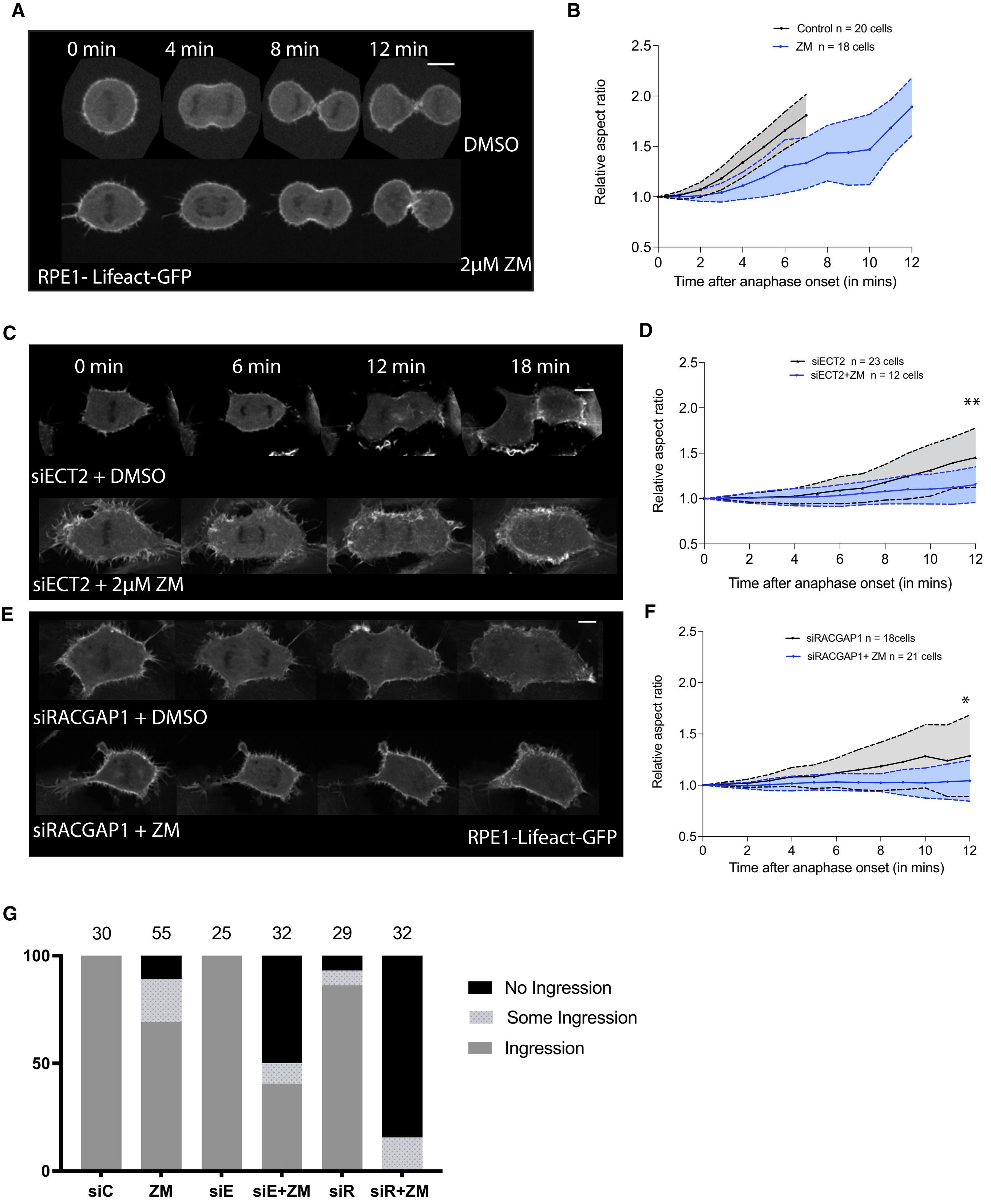
A
0 min
4 min
8 min
12 min
DMSO
2µM ZM
RPE1- Lifeact-GFP
B
Control n = 20 cells
ZM n = 18 cells
Relative aspect ratio
Time after anaphase onset (in mins)
C
0 min
6 min
12 min
18 min
siECT2 + DMSO
siECT2 + 2µM ZM
D
siECT2 n = 23 cells
siECT2+ZM n = 12 cells
**
Relative aspect ratio
Time after anaphase onset (in mins)
E
siRACGAP1 + DMSO
siRACGAP1 + ZM
RPE1-Lifeact-GFP
F
siRACGAP1 n = 18cells
siRACGAP1+ ZM n = 21 cells
*
Relative aspect ratio
Time after anaphase onset (in mins)
G
30
55
25
32
29
32
siC
ZM
siE
siE+ZM
siR
siR+ZM
No Ingression
Some Ingression
Ingression

Figure EV5.
