# Supplementary material for: Aurora B‐dependent polarization of the cortical actomyosin network during mitotic exit
Source: EMBO Rep. 2021 Aug 24;22(10):e52387. doi: 10.15252/embr.202152387 (PMC8490981; doi:10.15252/embr.202152387)
Supplement: Supplementary file 2 [file EMBR-22-e52387-s002.pdf]

EMBO reports

Aurora B-dependent polarization of the cortical actomyosin network during mitotic exit

Nitya Ramkumar, Jigna Patel, Jannis Anstatt, and Buzz Baum
DOI: 10.15252/embr.202152387

Corresponding author(s): Nitya Ramkumar (nitya.ramkumar@duke.edu)

Review Timeline:

Submission Date:	5th Jan 21
Editorial Decision:	3rd Feb 21
Revision Received:	10th May 21
Editorial Decision:	10th Jun 21
Revision Received:	15th Jul 21
Accepted:	26th Jul 21

Editor: Deniz Senyilmaz Tiebe

Transaction Report:

(Note: With the exception of the correction of typographical or spelling errors that could be a source of ambiguity, letters and reports are not edited. Depending on transfer agreements, referee reports obtained elsewhere may or may not be included in this compilation. Referee reports are anonymous unless the Referee chooses to sign their reports.)

1st Editorial Decision 3rd Feb 2021

Dear Dr. Ramkumar,

Thank you for submitting your manuscript to EMBO Reports. Three referees agreed to review your manuscript. So far, we have received two referee reports that are copied below. Given that both referees are in fair agreement that you should be given a chance to revise the manuscript, I would like to ask you to begin revising your study along the lines suggested by the referees.

Please note that this is a preliminary decision made in the interest of time, and that it is subject to change should the third referee offer very strong and convincing reasons for this. As soon as we receive the final report on your manuscript, we will forward it to you as well.

Referees express interest in the proposed role of Aurora B in polar relaxation at anaphase and actomyosin polarization during mitotic exit. However, they also raise important concerns that need to be addressed to consider publication here.

I find the reports informed and constructive, and believe that addressing the concerns raised will significantly strengthen the manuscript. As the reports are below, and I think all points need to be addressed, I will not detail them here.

Given these constructive comments, we would like to invite you to revise your manuscript with the understanding that the referee concerns (as in their reports) must be fully addressed and their suggestions taken on board. Please address all referee concerns in a complete point-by-point response. Acceptance of the manuscript will depend on a positive outcome of a second round of review. It is EMBO reports policy to allow a single round of revision only and acceptance or rejection of the manuscript will therefore depend on the completeness of your responses included in the next, final version of the manuscript.

We generally allow three months as standard revision time. As a matter of policy, competing manuscripts published during this period will not negatively impact on our assessment of the conceptual advance presented by your study. However, we request that you contact the editor as soon as possible upon publication of any related work, to discuss how to proceed. Should you foresee a problem in meeting this three-month deadline, please let us know in advance and we may be able to grant an extension.

*** Temporary update to EMBO Press scooping protection policy:
We are aware that many laboratories cannot function at full efficiency during the current COVID-19/SARS-CoV-2 pandemic and have therefore extended our 'scooping protection policy' to cover the period required for a full revision to address the experimental issues highlighted in the editorial decision letter. Please contact the scientific editor handling your manuscript to discuss a revision plan should you need additional time, and also if you see a paper with related content published elsewhere.***

IMPORTANT NOTE: we perform an initial quality control of all revised manuscripts before re-review. Your manuscript will FAIL this control and the handling will be DELAYED if the following APPLIES:
1. A data availability section providing access to data deposited in public databases is missing (where applicable).
2. Your manuscript contains statistics and error bars based on n=2. Please use scatter plots in these cases.

You can submit the revision either as a Scientific Report or as a Research Article. For Scientific Reports, the revised manuscript can contain up to 5 main figures and 5 Expanded View figures. If the revision leads to a manuscript with more than 5 main figures it will be published as a Research Article. In this case the Results and Discussion section can stay as it is now. If a Scientific Report is submitted, these sections have to be combined. This will help to shorten the manuscript text by eliminating some redundancy that is inevitable when discussing the same experiments twice. In either case, all materials and methods should be included in the main manuscript file.

Supplementary/additional data: The Expanded View format, which will be displayed in the main HTML of the paper in a collapsible format, has replaced the Supplementary information. You can submit up to 5 images as Expanded View. Please follow the nomenclature Figure EV1, Figure EV2 etc. The figure legend for these should be included in the main manuscript document file in a section called Expanded View Figure Legends after the main Figure Legends section. Additional Supplementary material should be supplied as a single pdf labeled Appendix. The Appendix includes a table of content on the first page with page numbers, all figures and their legends. Please follow the nomenclature Appendix Figure Sx throughout the text and also label the figures according to this nomenclature. For more details please refer to our guide to authors.

Please note that for all articles published beginning 1 July 2020, the EMBO Reports reference style will change to the Harvard style for all article types. Details and examples are provided at https://www.embopress.org/page/journal/14693178/authorguide#referencesformat

When submitting your revised manuscript, please carefully review the instructions that follow below. Failure to include requested items will delay the evaluation of your revision.

1) a .docx formatted version of the manuscript text (including legends for main figures, EV figures and tables). Please make sure that the changes are highlighted to be clearly visible.

2) individual production quality figure files as .eps, .tif, .jpg (one file per figure).

3) a .docx formatted letter INCLUDING the reviewers' reports and your detailed point-by-point responses to their comments. As part of the EMBO Press transparent editorial process, the point-by-point response is part of the Review Process File (RPF), which will be published alongside your paper. For more details on our Transparent Editorial Process, please visit our website: https://www.embopress.org/page/journal/14693178/authorguide#transparentprocess
You are able to opt out of this by letting the editorial office know (emboreports@embo.org). If you do opt out, the Review Process File link will point to the following statement: "No Review Process File is available with this article, as the authors have chosen not to make the review process public in this case."

4) a complete author checklist, which you can download from our author guidelines (<http://embor.embopress.org/authorguide>). Please insert information in the checklist that is also reflected in the manuscript. The completed author checklist will also be part of the RPF.

5) Please note that all corresponding authors are required to supply an ORCID ID for their name upon submission of a revised manuscript (<https://orcid.org/>). Please find instructions on how to link your ORCID ID to your account in our manuscript tracking system in our Author guidelines (<http://embor.embopress.org/authorguide>).

6) We replaced Supplementary Information with Expanded View (EV) Figures and Tables that are collapsible/expandable online. A maximum of 5 EV Figures can be typeset. EV Figures should be cited as 'Figure EV1, Figure EV2" etc... in the text and their respective legends should be included in the main text after the legends of regular figures.

- For the figures that you do NOT wish to display as Expanded View figures, they should be bundled together with their legends in a single PDF file called *Appendix*, which should start with a short Table of Content. Appendix figures should be referred to in the main text as: "Appendix Figure S1, Appendix Figure S2" etc. See detailed instructions regarding expanded view here: <http://embor.embopress.org/authorguide#expandedview>.

- Additional Tables/Datasets should be labeled and referred to as Table EV1, Dataset EV1, etc. Legends have to be provided in a separate tab in case of .xls files. Alternatively, the legend can be supplied as a separate text file (README) and zipped together with the Table/Dataset file.

7) We would also encourage you to include the source data for figure panels that show essential data.

Numerical data should be provided as individual .xls or .csv files (including a tab describing the data). For blots or microscopy, uncropped images should be submitted (using a zip archive if multiple images need to be supplied for one panel). Additional information on source data and instruction on how to label the files are available <http://embor.embopress.org/authorguide#sourcedata>.

8) Our journal encourages inclusion of *data citations in the reference list* to directly cite datasets that were re-used and obtained from public databases. Data citations in the article text are distinct from normal bibliographical citations and should directly link to the database records from which the data can be accessed. In the main text, data citations are formatted as follows: "Data ref: Smith et al, 2001" or "Data ref: NCBI Sequence Read Archive PRJNA342805, 2017". In the Reference list, data citations must be labeled with "[DATASET]". A data reference must provide the database name, accession number/identifiers and a resolvable link to the landing page from which the data can be accessed at the end of the reference. Further instructions are available at <http://embor.embopress.org/authorguide#datacitation>.

9) Please make sure to include a Data Availability Section before submitting your revision - if it is not applicable, make a statement that no data were deposited in a public database. Primary datasets (and computer code, where appropriate) produced in this study need to be deposited in an appropriate public database (see <http://embor.embopress.org/authorguide#dataavailability>).

Please remember to provide a reviewer password if the datasets are not yet public.

The accession numbers and database should be listed in a formal "Data Availability " section (placed after Materials & Method) that follows the model below. Please note that the Data Availability Section is restricted to new primary data that are part of this study.

Data availability

The datasets (and computer code) produced in this study are available in the following databases:

- RNA-Seq data: Gene Expression Omnibus GSE46843 (https://www.ncbi.nlm.nih.gov/geo/query/acc.cgi?acc=GSE46843)
- [data type]: [name of the resource] [accession number/identifier/doi] ([URL or identifiers.org/DATABASE:ACCESSION])

*** Note - All links should resolve to a page where the data can be accessed. ***

10) Regarding data quantification, please ensure to specify the name of the statistical test used to generate error bars and P values, the number (n) of independent experiments underlying each data point (not replicate measures of one sample), and the test used to calculate p-values in each figure legend. Discussion of statistical methodology can be reported in the materials and methods section, but figure legends should contain a basic description of n, P and the test applied.
Please note that error bars and statistical comparisons may only be applied to data obtained from at least three independent biological replicates.
Please also include scale bars in all microscopy images.

We would also welcome the submission of cover suggestions, or motifs to be used by our Graphics Illustrator in designing a cover.

I look forward to seeing a revised version of your manuscript when it is ready. Please let me know if you have questions or comments regarding the revision.

Kind regards,

Deniz Senyilmaz Tiebe

Deniz Senyilmaz Tiebe, PhD
Editor
EMBO Reports

Referee #1:

During cytokinesis, cells assemble an equatorial contractile ring that pinches the cell into two daughters. The generally round shape of the metaphase cell is transformed into an elongated shape as the two ends of the cell expand and move away from each other. How this process is regulated is not well understood. The process of polar relaxation contributes to cytokinesis by "relaxing" the polar cortices, often measured by the removal of F-actin and anillin at the two poles. Polar relaxation has a microtubule dependent component and also microtubule independent mechanism which is thought to involve signals from chromosomes.

In this manuscript, the authors test the role of two regulators of cytokinesis, centralspindlin and aurB kinase in driving cell shape changes in anaphase. They confirm that centralspindlin depletion (siRacGAP) prevents cytokinesis but not the cell shape changes observed in anaphase and the reduction in F actin. Similarly, inhibition of the CPC component aurora B kinase also prevents cytokinesis and blocks polarization of cells undergoing monopolar cytokinesis. Double inhibition, however, has a more profound effect, and cells remain rounded. This is an interesting observation; however, a mechanistic basis for this is not provided.

Comments:

1. The abundance of cortical Factin and the presence of actin filopodia, ruffles, blebs at the poles mean that it is not completely cleared in anaphase. A better description of how actin measurements were made - central slice from Z stack?- is needed. A line scan along the cortex (as done in fig 2) would show precisely where actin is cleared. Alternatively selecting a different probe (anillin? ERM?) for the bipolar cells is recommended. Measurement of aspect ratio for fig 1 would be good.

2. chromosomes have been shown to play a role in polar relaxation due to their proximilty to the cortex. The authors need to measure the distance of the chromosomes to the cortex under the various treatments; is the change in midzone microtubules following loss of CS or CPC changing chromosome position relative to cortex? What happens in cells that are doubly inhibited? Imaging the midzone microtubules would also be good (or referencing others that have done this).

3. Perhaps it is beyond the scope of the current work but using a probe that shows kinase activity would be a nice addition.

4. An aurA inhibitor is used on monopolar cytokinesis in suppl fig2 (8 cells) and the polarization is not blocked, as happens with ZM. additional controls would be nice to see, to be sure that ZM is acting as expected. Depletion of another CPC component would be another experimental approach to try.

5. the experiment in suppl Fig 4 should be expanded to show what happens to aspect ratio and cortical protein levels. The authors might also perform these expts (siEct2, with and without ZM treatment) on the hela cells, as the RPE cells can divide without the Rho activation.

Minor
Introduction reference in 3rd paragraph is wrong.

Supp 3 add control for top panel of A

Referee #3:

Ramkumar and colleagues present a concise and focussed manuscript reporting a role for Aurora B in clearance of DNA-proximal actin that is necessary for polar relaxation in dividing cells. The experiments are well performed, the data are clear and the interpretations made are sensible. The observations are novel, but mechanistically underdeveloped, which I think fit EMBO Reports' remit. As such, I am supportive of publication, with some minor changes as outlined below. I think most can be performed with textual changes:

1. The authors report in control cells: 'During this early anaphase, we observed a modest but reproducibly and statistically significant clearance of actin filaments from cell poles.', but I couldn't see the data or quantification that would permit this statement, nor is it clear to which panel the reader should look to find this data.

2. In the markup for Fig 1C, Aurora B is labelled as GFP, but I think it is mNG

3. In the RACGAP depleted cells (4D) mNG-AuroraB seems dimmer - is this consistent and are there any differences in Aurora B expression in the absence of centralspindlin components? Similarly, no change in RACGAP localisation is claimed in ZM-treated cells (4E), but there is much more RACGAP in the cytoplasm and the relative enrichment at spindle midzone is reduced. Is this not evidence of reciprocal communication between these components?

4. In figure 2B, the cortical polarisation and pERM-rich pseudofurrow is clear and correlates with the pseudofurrow forming on the DNA-distal pole of the cell. The failure of actin to clear from the DNA-proximal side in ZM-treated cells is subtle, but clear. The red dashed line in 2F makes seeing the quantification hard - could it underlay the data, rather than overlaying it?

5. Figure 3 is clear and convincing and evidences proximal DNA contributing to actin clearance in an Aurora B dependent manner using RAP1 overexpression to flatten the cell. It is perhaps worth commenting that the sphere-of-influence of the DNA-dependent actin clearance appears smaller in these RAP1 overexpressing cells - it is only a small DNA-proximal actin patch that is cleared, rather than a pole, as per the spherical monopolar assays. Stats on 3E are missing.

6. Statistical analysis, and the letters 'ngressuion' are missing from Figure S4C

7. Call outs for references, particularly those involving citation of book chapters, are improperly formatted (e.g., Rappaport & Cambridge University Press., 1996; Cytol & 1960)

Dear Dr. Ramkumar,

We have now received the report of referee #2 and I am copying it below as mentioned in my previous letter. Please respond to the comments of this referee as well during the revision.

I am looking forward to reading your revised manuscript when it is ready. Please contact us if you have any questions or comments about the revision.

Kind regards,

Deniz Senyilmaz Tiebe
--
Deniz Senyilmaz Tiebe, PhD
Editor
EMBO Reports

Referee #2:

Successful execution of cytokinesis, the last step of the cell division programme, requires the coordination of the tubulin and actin cytoskeleton with the membrane trafficking machinery, and many aspects of this are still incompletely understood. The mitotic kinase Aurora B has an important role in the regulation of the microtubule-based central spindle during anaphase and cytokinesis but also regulates other aspects of the process. Ramkumar and colleagues, here, specifically investigate the role of Aurora B in regulating actomyosin behaviour. They find that clearance of actomyosin from the poles during cytokinesis requires Aurora B activity, and that this an event that is independent of the presence of the central spindle. Furthermore, they show that the distance of the cortex to the chromatin seems to be relevant for actomyosin clearance suggesting an involvement of DNA derived signals. Lastly, the authors demonstrate that the combined inhibition of Aurora B activity and depletion of RacGAP1, important for central spindle formation, has the most drastic effect on cell shape changes during cytokinesis, in line with the idea that these two pathways act in parallel.
The main drawback of this work is that the manuscript is completely descriptive with no insight into the molecular biology underpinning the reported observations. Even the text is very vague, at multiple points in the text the authors refer to "DNA-dependent cues" without ever specifying or testing what this could be. The same applies for the role of Aurora B; it remains completely unclear whether the observed reliance of cortical actomyosin clearance on Aurora B is actually a direct effect and what possible targets of Aurora B may mediate this effect.
A further issue is that the manuscript is confusing to read because the authors start out with the reasonable hypothesis that Aurora B mediates cortical actomyosin clearance but then don't pursue this at a molecular level. Instead they move on to look at the effect of the distance of chromatin from the cortex, and then from there jump to the combined effect of Aurora B inhibition and RacGAP1 depletion. In the end, the reader is left with the feeling that none of these potentially important aspects have been satisfactorily addressed.

Minor points:
Canman et al., Nature, 2003 should be cited as the first paper to use a monopolar spindle system to look at the localisation of the chromosomal passenger proteins in the absence of a central spindle.
As the authors point out themselves, the central spindle is critical for cytokinesis, yet is never

visualised in the manuscript. For the correct interpretation of the events under investigation, it is important that there are no indirect effects on the microtubule cytoskeleton that may confound the analysis.

Referee #1:

During cytokinesis, cells assemble an equatorial contractile ring that pinches the cell into two daughters. The generally round shape of the metaphase cell is transformed into an elongated shape as the two ends of the cell expand and move away from each other. How this process is regulated is not well understood. The process of polar relaxation contributes to cytokinesis by "relaxing" the polar cortices, often measured by the removal of F-actin and anillin at the two poles. Polar relaxation has a microtubule dependent component and also microtubule independent mechanism which is thought to involve signals from chromosomes.

In this manuscript, the authors test the role of two regulators of cytokinesis, centralspindlin and aurB kinase in driving cell shape changes in anaphase. They confirm that centralspindlin depletion (siRacGAP) prevents cytokinesis but not the cell shape changes observed in anaphase and the reduction in F actin. Similarly, inhibition of the CPC component aurora B kinase also prevents cytokinesis and blocks polarization of cells undergoing monopolar cytokinesis. Double inhibition, however, has a more profound effect, and cells remain rounded.

This is a good summary of our main findings.

This is an interesting observation; however, a mechanistic basis for this is not provided.

In the revised manuscript we have strengthened our analysis. While the precise mechanistic details by which Aurora B acts remain to be resolved, we think our paper contributes to our understanding of this previously unappreciated role for Aurora B.

Comments:

1. The abundance of cortical Factin and the presence of actin filopodia, ruffles, blebs at the poles mean that it is not completely cleared in anaphase. A better description of how actin measurements were made - central slice from Z stack?- is needed. A line scan along the cortex (as done in fig 2) would show precisely where actin is cleared.

We thank the reviewer for their suggestion. As mentioned by the reviewer- we have used a projection of 2-z slices of the central region of the cell for our measurements and have amended our description to include this. We have also included line scans as suggested by the reviewer to better illustrate the loss of actin from polar regions in Figure 1 and Figure 5.

Alternatively selecting a different probe (anillin? ERM?) for the bipolar cells is recommended.

Use of an alternative probe is challenging as we would have to generate stable cell lines with live reporters to show relative clearance from metaphase to anaphase. The

transient nature of this clearance and the inability to synchronize the propagation of anaphase gives variable stages of mitotic exit, therefore giving confounding results.

Measurement of aspect ratio for fig 1 would be good.

-Aspect ratio for siRacgap is included in Fig 4E where we have compared cell shape changes in all treatment conditions.

2. chromosomes have been shown to play a role in polar relaxation due to their proximilty to the cortex. The authors need to measure the distance of the chromosomes to the cortex under the various treatments; is the change in midzone microtubules following loss of CS or CPC changing chromosome position relative to cortex?

We thank reviewer for this suggestion and have now included this quantification of chromatin position with different treatment conditions (Figure 5F-G). Proximity of chromatin to cortical actomyosin network remains unaffected upon loss of CS but is affected upon inhibition of CPC. This effect is enhanced in the double treatment.

What happens in cells that are doubly inhibited? Imaging the midzone microtubules would also be good (or referencing others that have done this).

We thank the reviewer for this suggestion and have included a panel showing midzone microtubules and midzone PLK1 accumulation with different treatments (Figure 4C, EV4A)

3. Perhaps it is beyond the scope of the current work but using a probe that shows kinase activity would be a nice addition.

We thank the reviewer for their suggestion. We tried to use membrane targeted Aurora B-FRET sensor to assess the activity of the kinase. In order to visualize region specific Aurora B activity, the sensor needs to be targeted to– centromere, microtubule, membrane, etc. as the cytoplasmic FRET sensor is unable to recapitulate the tagged sensor activity. Owing to the dynamic re-localization of Aurora B, we found the results not very useful for this study.

4. An aurA inhibitor is used on monopolar cytokinesis in suppl fig2 (8 cells) and the polarization is not blocked, as happens with ZM. additional controls would be nice to see, to be sure that ZM is acting as expected. Depletion of another CPC component would be another experimental approach to try.

We thank the reviewer for this suggestion. We have included knockdown of INCENP, a core member of the CPC complex and performed our monopolar exit analysis. Similar to MKLP2 knockdown, we find that INCENP knockdown also leads to a loss in polarization of pERM during monopolar exit.

5. the experiment in suppl Fig 4 should be expanded to show what happens to aspect

ratio and cortical protein levels. The authors might also perform these expts (siEct2, with and without ZM treatment) on the hela cells, as the RPE cells can divide without the Rho activation.

We thank the reviewer for their suggestions.
1. We have now expanded the figure to include the cell shape quantifications for RPE1 cells under different treatment (Figure S5).
RPE1 cells are migratory cells and therefore, unlike Hela cells, the lamellipodia and other actin dependent projections make is harder to definitively quantify cortical protein levels during anaphase. However, we think cell shape changes, quantified as aspect ratio is a good indicator of cell polarization.

2. We have also included data on Hela cells treated with siEct2 and siEct2+ZM (Figure S4). Similar to RPE1 cells, double treatment of cells with siEct2 and ZM completely abrogates any shape changes.

Minor
Introduction reference in 3rd paragraph is wrong.

We thank the reviewer and have amended the reference.

Supp 3 add control for top panel of A

We have corrected the figure to reflect this.

Referee #3:

Ramkumar and colleagues present a concise and focussed manuscript reporting a role for Aurora B in clearance of DNA-proximal actin that is necessary for polar relaxation in dividing cells. The experiments are well performed, the data are clear and the interpretations made are sensible. The observations are novel, but mechanistically underdeveloped, which I think fit EMBO Reports' remit. As such, I am supportive of publication, with some minor changes as outlined below. I think most can be performed with textual changes:

We thank the reviewer for their assessment.

1. The authors report in control cells: 'During this early anaphase, we observed a modest but reproducibly and statistically significant clearance of actin filaments from cell poles.', but I couldn't see the data or quantification that would permit this statement, nor is it clear to which panel the reader should look to find this data.

We thank the reviewer for their suggestion and have amended the text to reflect this. We have also included statistical analysis to show that there is significant reduction of actin levels in both siControl and siRacGAP treated cells. (Figure1)

We amended the text - *“During this early anaphase, we observed a modest but significant clearance of actin filaments from cell poles (Figure 1A-E) as seen by actin intensity levels clustering below 1 (normalized to metaphase levels).”*

We have also added line scan plots to better illustrate the loss of actin filaments from the poles.

2. In the markup for Fig 1C, Aurora B is labelled as GFP, but I think it is mNG

We have amended the figure.

3. In the RACGAP depleted cells (4D) mNG-AuroraB seems dimmer - is this consistent and are there any differences in Aurora B expression in the absence of centralspindlin components?

We thank the reviewer for their suggestion. We quantified the levels of Aurora B upon centralspindlin depletion and found a moderate decrease in its accumulation at the midzone. We have included a figure panel to reflect this quantification (Figure S1D). However, we do not see a correlation between the level of Aurora B accumulation at the spindle midzone and the ability of cells to perform cytokinesis. Irrespective of the levels of Aurora B at the midzone, the cells fail to form a furrow.

Similarly, no change in RACGAP localisation is claimed in ZM-treated cells (4E), but there is much more RACGAP in the cytoplasm and the relative enrichment at spindle midzone is reduced. Is this not evidence of reciprocal communication between these components?

We thank the reviewer for their careful observation. We have amended the text to reflect this.

4. In figure 2B, the cortical polarisation and pERM-rich pseudofurrow is clear and correlates with the pseudofurrow forming on the DNA-distal pole of the cell. The failure of actin to clear from the DNA-proximal side in ZM-treated cells is subtle, but clear. The red dashed line in 2F makes seeing the quantification hard - could it underlay the data, rather than overlaying it?

We thank the reviewer for this suggestion and have moved the dashed line as an underlay.

5. Figure 3 is clear and convincing and evidences proximal DNA contributing to actin clearance in an Aurora B dependent manner using RAP1 overexpression to flatten the cell. It is perhaps worth commenting that the sphere-of-influence of the DNA-dependent actin clearance appears smaller in these RAP1 overexpressing cells - it is only a small DNA-proximal actin patch that is cleared, rather than a pole, as per the spherical

monopolar assays. Stats on 3E are missing.

We thank the reviewer for this suggestion. The reviewer is correct in pointing out that the effect of DNA on the relaxation is localized to the region directly in close proximity to it. It perhaps works in a similar manner in the polar regions. We currently do not have the ability to splay out the polar region to visualize this local effect and therefore in the tangential view, visualize it as a gradient.

We had included statistical analysis - Chi-square test to compare distribution of cells in Figure 3E and Figure EV3B, both of which show significant difference.

6. Statistical analysis, and the letters 'ngressuion' are missing from Figure S4C

We have amended the figure and also included statistical analysis for Figure EV4C, currently Figure EV5G. Chi-square test shows significant difference.

7. Call outs for references, particularly those involving citation of book chapters, are improperly formatted (e.g., Rappaport & Cambridge University Press., 1996; Cytol & 1960)

We have amended the text accordingly.

Reviewer 2

Successful execution of cytokinesis, the last step of the cell division programme, requires the coordination of the tubulin and actin cytoskeleton with the membrane trafficking machinery, and many aspects of this are still incompletely understood. The mitotic kinase Aurora B has an important role in the regulation of the microtubule-based central spindle during anaphase and cytokinesis but also regulates other aspects of the process. Ramkumar and colleagues, here, specifically investigate the role of Aurora B in regulating actomyosin behaviour. They find that clearance of actomyosin from the poles during cytokinesis requires Aurora B activity, and that this an event that is independent of the presence of the central spindle. Furthermore, they show that the distance of the cortex to the chromatin seems to be relevant for actomyosin clearance suggesting an involvement of DNA derived signals. Lastly, the authors demonstrate that the combined inhibition of Aurora B activity and depletion of RacGAP1, important for central spindle formation, has the most drastic effect on cell shape changes during cytokinesis, in line with the idea that these two pathways act in parallel.

This is a good summary of our work.

The main drawback of this work is that the manuscript is completely descriptive with no insight into the molecular biology underpinning the reported observations. Even the text is very vague, at multiple points in the text the authors refer to "DNA-dependent cues" without ever specifying or testing what this could be. The same applies for the role of Aurora B; it remains completely unclear whether the observed reliance of cortical

actomyosin clearance on Aurora B is actually a direct effect and what possible targets of Aurora B may mediate this effect.

We respectfully disagree that this is “completely descriptive”. We describe a causal mechanistic role for Aurora B in polar relaxation. We would note that there are almost no systems in which we know the complete molecular and biophysical processes that underpin division.

A further issue is that the manuscript is confusing to read because the authors start out with the reasonable hypothesis that Aurora B mediates cortical actomyosin clearance but then don't pursue this at a molecular level. Instead they move on to look at the effect of the distance of chromatin from the cortex, and then from there jump to the combined effect of Aurora B inhibition and RacGAP1 depletion. In the end, the reader is left with the feeling that none of these potentially important aspects have been satisfactorily addressed.

Our work addresses an important cell biological problem - anaphase polarization. Successful completion of cytokinesis requires both the formation of a contractile actomyosin ring and the softening of the polar region. Using a range of perturbations - cell shape, monopolar spindles, small molecular inhibition and RNAi, we identify a novel role for Aurora B, in regulating polar relaxation. This function of Aurora B is independent of its role in regulating centralspindlin. Therefore, Aurora B emerges as a central player in cytokinesis by coordinating the changes in actomyosin at the equatorial and polar regions to ensure a successful cytokinesis. While much more work needs to be done to identify the precise molecular details by which Aurora B executes this function – our work helps open up a new avenue of research that we believe will help to stimulate research into this understudied aspect of cytokinesis.

Minor points:
Canman et al., Nature, 2003 should be cited as the first paper to use a monopolar spindle system to look at the localisation of the chromosomal passenger proteins in the absence of a central spindle.

We have amended the text accordingly and referenced the paper.

As the authors point out themselves, the central spindle is critical for cytokinesis, yet is never visualised in the manuscript. For the correct interpretation of the events under investigation, it is important that there are no indirect effects on the microtubule cytoskeleton that may confound the analysis.

We thank the reviewer for this suggestion and have now included two figure panels showing immunostaining of microtubules (Figure 4C) and spindle midzone component PLK1 (Figure EV4A). They both show that midzone is formed normally in early anaphase upon RacGAP knockdown, but fails to be stabilized soon after. In contrast, ZM treatment leads to a disperse midzone which persists through mitotic exit. In the double treatment, there is no recognizable spindle midzone.

We demonstrate that cells are able to clear actin from their poles under various treatments where the midzone is altered -
-upon RacGAP knockdown- where the midzone is unstable,
-in monopolar cytokinesis, where there is no over-lapping microtubules or spindle midzone structure.
-upon RacGAP knockdown during monopolar cytokinesis.

This suggests that the ability of cells to relax their poles must be independent of the function of central spindle during cytokinesis.

1st Revision - Editorial Decision 10th Jun 2021

Dear Dr. Ramkumar,

Thank you for submitting your revised manuscript. It has now been seen by two of the original referees.

As you can see, the referee finds that the study is significantly improved during revision and recommends publication. However, I need you to address the editorial points below before I can accept the manuscript.

• Please address the remaining minor concerns of referee #1.
• As per our format requirements, manuscripts containing 5 main figures are classified as 'reports', however, your manuscript format is 'article'. Therefore, please convert your manuscript format into a 'report' and thus combine the results and the discussion sections. Please see https://www.embopress.org/page/journal/14693178/authorguide#researcharticleguide
• Please provide 3-5 keywords for your study. These will be visible in the html version of the paper and on PubMed and will help increase the discoverability of your work.
• As per our guidelines, please add a 'Data Availability Section', where you state that no data were deposited in a public database.
• Please rename the 'Author statement' section as 'Conflict of Interest'.
• Please rename the 'Methods' section as 'Materials and Methods'
• Please reorder the manuscript sections in the order shown here: https://www.embopress.org/page/journal/14693178/authorguide#textformat
• Please include the funding information also in the manuscript text - i.e., in the Acknowledgements section.
• We note that EV2C-D are currently not called out in the text.
• We realize EV Figures are currently called as supplementary figures in the figure legends and in the figure files, which need to be corrected to EV Figure #.
• Papers published in EMBO Reports include a 'synopsis' and 'bullet points' to further enhance discoverability. Both are displayed on the html version of the paper and are freely accessible to all readers. The synopsis includes a short standfirst summarizing the study in 1 or 2 sentences that summarize the paper and are provided by the authors and streamlined by the handling editor. I would therefore ask you to include your synopsis blurb and 3-5 bullet points listing the key experimental findings.
• In addition, please provide an image for the synopsis. This image should provide a rapid overview of the question addressed in the study but still needs to be kept fairly modest since the image size cannot exceed 550x400 pixels.
• Our production/data editors have asked you to clarify several points in the figure legends (see attached document). Please incorporate these changes in the attached word document and return it with track changes activated.

Thank you again for giving us to consider your manuscript for EMBO Reports, I look forward to your minor revision.

Kind regards,

Deniz Senyilmaz Tiebe

--
Deniz Senyilmaz Tiebe, PhD
Editor
EMBO Reports

Referee #1:

The authors have responded to the reviewer's concerns. I have a few remaining issues, that the authors should address prior to publication.

In the Figures the authors show the actin (or ERM) fluorescence intensity around the cell cortex by graphing distance around the cortex vs the relative intensity (see for example figure 2). In other cases (i.e., Figure 5) they show a single trace of normalized intensity vs normalized distance. The first method does a much better job of showing the trend around the cell, as compared with the single lines (see fig 5E). Can the authors please use the average of several cells instead of the single trace?

The authors provide data consistent with a role for aurora B activity in cell polarization and cortical actin clearance during the events of late anaphase. They do not cite Mangal JCB 2018 who showed that clearance of anillin from the polar cortex requires Aurora A activation by TPXl1 in C. elegans. The current Ms shows that a chemical inhibitor of Aurora A has no effect on cortical polarization. The authors should cite Mangal et al and add a statement of the reasons that might give rise to the different results.

On page 5 the authors mention that depletion of MKLP2 prevented pERM cortical polarization "suggesting a role for Aurora B localization and activity". MKLP2 should alter the localization of the CPC to the midzone, but not AurB activity, so one might expect that loss of MKLP2 would not show the same effect as ZM. Please clarify.

In Figure 5, quantification in C. please show the quantification for the siR and DMSO on the graph.

Referee #3:

My concerns have been addressed - all looks fine to me

We thank the reviewer for their comments and have revised the manuscript to include the suggestions.

In the Figures the authors show the actin (or ERM) fluorescence intensity around the cell cortex by graphing distance around the cortex vs the relative intensity (see for example figure 2). In other cases (i.e., Figure 5) they show a single trace of normalized intensity vs normalized distance. The first method does a much better job of showing the trend around the cell, as compared with the single lines (see fig 5E). Can the authors please use the average of several cells instead of the single trace?

We thank the reviewer for their suggestion. We have replaced graphs for single cells with graphs showing the trend for average population of cells under different treatments (Figure 5E).

The authors provide data consistent with a role for aurora B activity in cell polarization and cortical actin clearance during the events of late anaphase. They do not cite Mangal JCB 2018 who showed that clearance of anillin from the polar cortex requires Aurora A activation by TPXl1 in C. elegans. The current Ms shows that a chemical inhibitor of Aurora A has no effect on cortical polarization. The authors should cite Mangal et al and add a statement of the reasons that might give rise to the different results.

We thank the reviewer for their suggestion and have included this in our discussion.

“Although we did not observe a defect in DNA-dependent clearance upon Aurora A inhibition in human cells (Figure EV2A-B), Aurora A has been identified as an aster-based signal for facilitating polar relaxation in the *C. elegans* zygote (Mangal *et al*, 2018). Since the two homologous Aurora kinases have a largely similar kinase domain, their function may largely reflect their different dynamic sub-cellular localization (Li *et al*, 2015; Fu *et al*, 2009).”

On page 5 the authors mention that depletion of MKLP2 prevented pERM cortical polarization "suggesting a role for Aurora B localization and activity". MKLP2 should alter the localization of the CPC to the midzone, but not AurB activity, so one might expect that loss of MKLP2 would not show the same effect as ZM. Please clarify.

Our data with MKLP2 silencing suggests that Aurora B activity needs to be spatially regulated to induce polar relaxation. Therefor we hypothesize that the switch in Aurora B localization from centromeres to midzone at early anaphase controls the spatial region of its activity which in turn regulates polar relaxation. We have amended the text to reflect this.
“Interestingly, cortical polarization was affected in a similar way by depletion of MKLP2 (Figure EV2C-D), a kinesin required for Aurora B re-localization during anaphase (Serena *et al*, 2015). This suggests a role for spatial control of Aurora B activity in polarizing the cortex at mitotic exit.”

In Figure 5, quantification in C. please show the quantification for the siR and DMSO on the graph.

We have amended Figure 5C to include the above conditions.

2nd Revision - Editorial Decision 26th Jul 2021

Dear Dr. Ramkumar,

Thank you for submitting your revised manuscript. I have now looked at everything and all is fine. Therefore, I am very pleased to accept your manuscript for publication in EMBO Reports.

Congratulations on a nice work!

Kind regards,

Deniz Senyilmaz Tiebe
--
Deniz Senyilmaz Tiebe, PhD
Editor
EMBO Reports

--

At the end of this email I include important information about how to proceed. Please ensure that you take the time to read the information and complete and return the necessary forms to allow us to publish your manuscript as quickly as possible.

As part of the EMBO publication's Transparent Editorial Process, EMBO reports publishes online a Review Process File to accompany accepted manuscripts. As you are aware, this File will be published in conjunction with your paper and will include the referee reports, your point-by-point response and all pertinent correspondence relating to the manuscript.

If you do NOT want this File to be published, please inform the editorial office within 2 days, if you have not done so already, otherwise the File will be published by default [contact: emboreports@embo.org]. If you do opt out, the Review Process File link will point to the following statement: "No Review Process File is available with this article, as the authors have chosen not to make the review process public in this case."

Should you be planning a Press Release on your article, please get in contact with emboreports@wiley.com as early as possible, in order to coordinate publication and release dates.

Thank you again for your contribution to EMBO reports and congratulations on a successful publication. Please consider us again in the future for your most exciting work.

****

THINGS TO DO NOW:

You will receive proofs by e-mail approximately 2-3 weeks after all relevant files have been sent to our Production Office; you should return your corrections within 2 days of receiving the proofs.

Please inform us if there is likely to be any difficulty in reaching you at the above address at that time. Failure to meet our deadlines may result in a delay of publication, or publication without your

corrections.

All further communications concerning your paper should quote reference number EMBOR-2021-52387V3 and be addressed to emboreports@wiley.com.

Should you be planning a Press Release on your article, please get in contact with emboreports@wiley.com as early as possible, in order to coordinate publication and release dates.

EMBO PRESS

YOU MUST COMPLETE ALL CELLS WITH A PINK BACKGROUND ↓

PLEASE NOTE THAT THIS CHECKLIST WILL BE PUBLISHED ALONGSIDE YOUR PAPER

Corresponding Author Name: Nitya Ramkumar, Buzz Baum
Journal Submitted to: EMBO reports
Manuscript Number: EMBOR-2021-52387V2

Reporting Checklist For Life Sciences Articles (Rev. June 2017)

This checklist is used to ensure good reporting standards and to improve the reproducibility of published results. These guidelines are consistent with the Principles and Guidelines for Reporting Preclinical Research issued by the NIH in 2014. Please follow the journal's authorship guidelines in preparing your manuscript.

A- Figures

1. Data

The data shown in figures should satisfy the following conditions:

- the data were obtained and processed according to the field's best practice and are presented to reflect the results of the experiments in an accurate and unbiased manner.
- figure panels include only data points, measurements or observations that can be compared to each other in a scientifically meaningful way.
- graphs include clearly labeled error bars for independent experiments and sample sizes. Unless justified, error bars should not be shown for technical replicates.
- if n< 5, the individual data points from each experiment should be plotted and any statistical test employed should be justified
- Source Data should be included to report the data underlying graphs. Please follow the guidelines set out in the author ship guidelines on Data Presentation.

2. Captions

Each figure caption should contain the following information, for each panel where they are relevant:

- a specification of the experimental system investigated (eg cell line, species name).
- the assay(s) and method(s) used to carry out the reported observations and measurements
- an explicit mention of the biological and chemical entity(ies) that are being measured.
- an explicit mention of the biological and chemical entity(ies) that are altered/varied/perturbed in a controlled manner.

- the exact sample size (n) for each experimental group/condition, given as a number, not a range;
- a description of the sample collection allowing the reader to understand whether the samples represent technical or biological replicates (including how many animals, litters, cultures, etc.).
- a statement of how many times the experiment shown was independently replicated in the laboratory.
- definitions of statistical methods and measures:
  - common tests, such as t-test (please specify whether paired vs. unpaired), simple $\chi 2$ tests, Wilcoxon and Mann-Whitney tests, can be unambiguously identified by name only, but more complex techniques should be described in the methods section;
  - are tests one-sided or two-sided?
  - are there adjustments for multiple comparisons?
  - exact statistical test results, e.g., P values = x but not P values < x;
  - definition of 'center values' as median or average;
  - definition of error bars as s.d. or s.e.m.

Any descriptions too long for the figure legend should be included in the methods section and/or with the source data.

In the pink boxes below, please ensure that the answers to the following questions are reported in the manuscript itself. Every question should be answered. If the question is not relevant to your research, please write NA (non applicable). We encourage you to include a specific subsection in the methods section for statistics, reagents, animal models and human subjects.

USEFUL LINKS FOR COMPLETING THIS FORM

http://www.antibodypedia.com
http://1degreebio.org
http://www.equator-network.org/reporting-guidelines/improving-bioscience-research-report
http://grants.nih.gov/grants/olaw/olaw.htm
http://www.mrc.ac.uk/Ourresearch/Ethicsresearchguidance/Useofanimals/index.htm
http://ClinicalTrials.gov
http://www.consort-statement.org
http://www.consort-statement.org/checklists/view/32-consort/66-title
http://www.equator-network.org/reporting-guidelines/reporting-recommendations-for-tumo
http://datadryad.org
http://figshare.com
http://www.ncbi.nlm.nih.gov/gap
http://www.ebi.ac.uk/ega
http://biomodels.net/
http://biomodels.net/miriam/
http://jjj.biochem.sun.ac.za
http://oba.od.nih.gov/biosecurity/biosecurity_documents.html
http://www.selectagents.gov/

B- Statistics and general methods

Please fill out these boxes ↓ (Do not worry if you cannot see all your text once you press return)

1.a. How was the sample size chosen to ensure adequate power to detect a pre-specified effect size?	Each experiment was performed a minimum of 3 independent times
1.b. For animal studies, include a statement about sample size estimate even if no statistical methods were used.	NA
2. Describe inclusion/exclusion criteria if samples or animals were excluded from the analysis. Were the criteria pre-established?	All samples that were imaged or stained were included in the anaysis.
3. Were any steps taken to minimize the effects of subjective bias when allocating animals/samples to treatment (e.g. randomization procedure)? If yes, please describe.	All samples were treated equally
For animal studies, include a statement about randomization even if no randomization was used.	NA
4.a. Were any steps taken to minimize the effects of subjective bias during group allocation or/and when assessing results (e.g. blinding of the investigator)? If yes please describe.	NA
4.b. For animal studies, include a statement about blinding even if no blinding was done	NA
5. For every figure, are statistical tests justified as appropriate?	Yes. The description of the test and p-values are mentioned in respective figure legends.
Do the data meet the assumptions of the tests (e.g., normal distribution)? Describe any methods used to assess it.	Yes, they are described in the figure legend.
Is there an estimate of variation within each group of data?	Standard deviations are shown in all graphs.

Is the variance similar between the groups that are being statistically compared?	They are described in the figure legend.

C- Reagents

6. To show that antibodies were profiled for use in the system under study (assay and species), provide a citation, catalog number and/or clone number, supplementary information or reference to an antibody validation profile. e.g., Antibodypedia (see link list at top right), 1DegreeBio (see link list at top right).	pERM (Cell signaling 3726), pMLC (Cell Signaling 3671), RACGAP1 (Everest Biotech), Aurora B (Abcam 2254), Anillin (gift from C.Field), PLK1 (Cell Signalling 4513) and tubulin (Sigma T9026)
7. Identify the source of cell lines and report if they were recently authenticated (e.g., by STR profiling) and tested for mycoplasma contamination.	Hela Kyoto, Hela-13, Hela-Cdk1as, RPE1-Lifeact. Cell lines were routinely tested for myocoplasma contamination

* for all hyperlinks, please see the table at the top right of the document

D- Animal Models

8. Report species, strain, gender, age of animals and genetic modification status where applicable. Please detail housing and husbandry conditions and the source of animals.	NA
9. For experiments involving live vertebrates, include a statement of compliance with ethical regulations and identify the committee(s) approving the experiments.	NA
10. We recommend consulting the ARRIVE guidelines (see link list at top right) (PLoS Biol. 8(6), e1000412, 2010) to ensure that other relevant aspects of animal studies are adequately reported. See author guidelines, under 'Reporting Guidelines'. See also: NIH (see link list at top right) and MRC (see link list at top right) recommendations. Please confirm compliance.	NA

E- Human Subjects

11. Identify the committee(s) approving the study protocol.	NA
12. Include a statement confirming that informed consent was obtained from all subjects and that the experiments conformed to the principles set out in the WMA Declaration of Helsinki and the Department of Health and Human Services Belmont Report.	NA
13. For publication of patient photos, include a statement confirming that consent to publish was obtained.	NA
14. Report any restrictions on the availability (and/or on the use) of human data or samples.	NA
15. Report the clinical trial registration number (at ClinicalTrials.gov or equivalent), where applicable.	NA
16. For phase II and III randomized controlled trials, please refer to the CONSORT flow diagram (see link list at top right) and submit the CONSORT checklist (see link list at top right) with your submission. See author guidelines, under 'Reporting Guidelines'. Please confirm you have submitted this list.	NA
17. For tumor marker prognostic studies, we recommend that you follow the REMARK reporting guidelines (see link list at top right). See author guidelines, under 'Reporting Guidelines'. Please confirm you have followed these guidelines.	NA

F- Data Accessibility

18: Provide a "Data Availability" section at the end of the Materials & Methods, listing the accession codes for data generated in this study and deposited in a public database (e.g. RNA-Seq data: Gene Expression Omnibus GSE39462, Proteomics data: PRIDE PXD000208 etc.) Please refer to our author guidelines for 'Data Deposition'.<br><br>Data deposition in a public repository is mandatory for:<br>a. Protein, DNA and RNA sequences<br>b. Macromolecular structures<br>c. Crystallographic data for small molecules<br>d. Functional genomics data<br>e. Proteomics and molecular interactions	NA
19. Deposition is strongly recommended for any datasets that are central and integral to the study; please consider the journal's data policy. If no structured public repository exists for a given data type, we encourage the provision of datasets in the manuscript as a Supplementary Document (see author guidelines under 'Expanded View' or in unstructured repositories such as Dryad (see link list at top right) or Figshare (see link list at top right).	NA
20. Access to human clinical and genomic datasets should be provided with as few restrictions as possible while respecting ethical obligations to the patients and relevant medical and legal issues. If practically possible and compatible with the individual consent agreement used in the study, such data should be deposited in one of the major public access-controlled repositories such as dbGAP (see link list at top right) or EGA (see link list at top right).	NA
21. Computational models that are central and integral to a study should be shared without restrictions and provided in a machine-readable form. The relevant accession numbers or links should be provided. When possible, standardized format (SBML, CellML) should be used instead of scripts (e.g. MATLAB). Authors are strongly encouraged to follow the MIRIAM guidelines (see link list at top right) and deposit their model in a public database such as Biomodels (see link list at top right) or JWS Online (see link list at top right). If computer source code is provided with the paper, it should be deposited in a public repository or included in supplementary information.	NA

G- Dual use research of concern

22. Could your study fall under dual use research restrictions? Please check biosecurity documents (see link list at top right) and list of select agents and toxins (APHIS/CDC) (see link list at top right). According to our biosecurity guidelines, provide a statement only if it could.	NA
